# Substantial Histone Reduction Modulates Genomewide Nucleosomal Occupancy and Global Transcriptional Output

**DOI:** 10.1371/journal.pbio.1001086

**Published:** 2011-06-28

**Authors:** Barbara Celona, Assaf Weiner, Francesca Di Felice, Francesco M. Mancuso, Elisa Cesarini, Riccardo L. Rossi, Lorna Gregory, Dilair Baban, Grazisa Rossetti, Paolo Grianti, Massimiliano Pagani, Tiziana Bonaldi, Jiannis Ragoussis, Nir Friedman, Giorgio Camilloni, Marco E. Bianchi, Alessandra Agresti

**Affiliations:** 1 San Raffaele University, Milan, Italy; 2 School of Computer Science and Engineering, Hebrew University, Jerusalem, Israel; 3 Alexander Silberman Institute of Life Sciences, Hebrew University, Jerusalem, Israel; 4 Dipartimento di Biologia e Biotecnologie, Università di Roma La Sapienza, Rome, Italy; 5 IFOM-IEO Campus, Milan, Italy; 6 Integrative Biology Program, Istituto Nazionale di Genetica Molecolare, Milan, Italy; 7 Wellcome Trust Centre for Human Genetics, University of Oxford, Oxford, United Kingdom; 8 Dipartimento di Scienze Biomolecolari e Biotecnologie, Università degli Studi di Milano, Milan, Italy; 9 Istituto di Biologia e Patologia Molecolari, CNR, Rome, Italy; 10 Division of Genetics and Cell Biology, San Raffaele Research Institute, Milan, Italy; Adolf Butenandt Institute, Germany

## Abstract

The basic unit of genome packaging is the nucleosome, and nucleosomes have long been proposed to restrict DNA accessibility both to damage and to transcription. Nucleosome number in cells was considered fixed, but recently aging yeast and mammalian cells were shown to contain fewer nucleosomes. We show here that mammalian cells lacking High Mobility Group Box 1 protein (HMGB1) contain a reduced amount of core, linker, and variant histones, and a correspondingly reduced number of nucleosomes, possibly because HMGB1 facilitates nucleosome assembly. Yeast *nhp6* mutants lacking Nhp6a and -b proteins, which are related to HMGB1, also have a reduced amount of histones and fewer nucleosomes. Nucleosome limitation in both mammalian and yeast cells increases the sensitivity of DNA to damage, increases transcription globally, and affects the relative expression of about 10% of genes. In yeast *nhp6* cells the loss of more than one nucleosome in four does not affect the location of nucleosomes and their spacing, but nucleosomal occupancy. The decrease in nucleosomal occupancy is non-uniform and can be modelled assuming that different nucleosomal sites compete for available histones. Sites with a high propensity to occupation are almost always packaged into nucleosomes both in wild type and nucleosome-depleted cells; nucleosomes on sites with low propensity to occupation are disproportionately lost in nucleosome-depleted cells. We suggest that variation in nucleosome number, by affecting nucleosomal occupancy both genomewide and gene-specifically, constitutes a novel layer of epigenetic regulation.

## Author Summary

The accurate preservation and correct retrieval of genetic information is crucial for all living organisms. In eukaryotes, whether single-celled yeast or complex mammals, the DNA containing the genetic information is wrapped around beads of histone proteins to form structures called nucleosomes along the length of the DNA; this packaging arrangement helps protect the genome from damage and may restrict access to the genetic information. Until recently, the amount of histones and, consequently, the number of nucleosomes in the cell were considered fixed. Here, we show that in both mammalian and yeast cells that lack a single protein—HMGB1 in mammals or Nhp6a/b in yeast—the abundance of histones and nucleosomes decreases by 20%–30%. Contrary to expectations, we found that in yeast the nucleosomes do not redistribute along DNA when they are fewer: they largely maintain their positions, but the amount of time each specific DNA site spends wrapped in a nucleosome (i.e., its occupancy) decreases. Sequences that are already less frequently occupied than others in normal yeast cells lose disproportionally more nucleosomes in the mutant yeast that lack Nhp6a/b. This gives rise to a global increase in transcription and specific alterations in the expression of certain genes. This study thus contributes to a deeper understanding of how the DNA is packaged and organized. It also suggests that the cell's histone content might contribute in an important way to gene regulation.

## Introduction

In eukaryotic cells, genetic information is organized in chromatin, a highly conserved structural polymer of DNA and histones whose basic unit is the nucleosome [1]. Dynamic changes in the local or global organization of chromatin are required in order to perform most nuclear activities, including replication, transcription, and DNA repair [2],[3]. Maintenance of such a dynamic structure, in terms of spatial distribution of nucleosomes and proper reorganization during nuclear activities, is considered crucial to preserve cellular identity and to protect cells from genomic instabilities that are among the major causative factors in aging and cancer. Until recently, no gross modifications of nucleosome number in cells were described or even looked for, even if differences in nucleosome linker length were observed between different cell types [4]. However, recent work has showed that aging yeast [5] and mammalian [6] cells contain fewer nucleosomes. We show here that mammalian cells lacking High Mobility Group Box 1 (HMGB1) protein contain a substantially reduced amount of histones and nucleosomes. Yeast cells lacking Nhp6a/Nhp6b proteins, which are functionally similar to HMGB1 [7], have a very similar phenotype, suggesting that the involvement of HMG-box proteins in controlling nucleosome number is conserved in evolution.

HMGB1 is an abundant non-histone chromatin protein that binds to the minor groove of DNA without sequence specificity and, to a large number of nuclear proteins, contributing to the maintenance, retrieval, and expression of genetic information [8]. HMGB1 is composed by two DNA binding domains, called HMG-boxes, followed by a long unstructured tail that appears to modulate the interaction of the HMG-boxes with DNA [9]. HMGB1 binds to nucleosomes at the dyad axis and appears to compete with histone H1, exerting opposite effects: HMGB1 facilitates nucleosome sliding and makes chromatin more accessible, H1 restrains nucleosome sliding and makes chromatin less accessible [10],[11]. *Hmgb1*−/− mice die soon after birth with a complex, pleiotropic phenotype [12]. Yeast cells contain two abundant HMG-box proteins, called Nhp6a and Nhp6b, which are composed of a single, non-sequence-specific HMG-box and are functionally redundant, since the loss of only one of the two Nhp6 proteins causes a very mild phenotype.

Both mammalian cells lacking HMGB1 and yeast cells lacking both Nhp6a and -b are viable, although they display a number of defects [12],[13]. Specifically, the *nhp6a/b* double mutant (henceforth *nhp6*) yeast cells and *Hmgb1*−/− MEFs display genomic instability and hypersensitivity to DNA-damaging agents; *nhp6* cells have a shorter life span and increased levels of extrachromosomal rDNA circles (a hallmark of senescence) [14]. Curiously, in both mammalian and yeast mutants, a given dose of UV irradiation appeared to produce almost twice as many thymidine dimers as in wild type cells [14]. We show here that these cells are also more sensitive to ionizing radiation, which is due to a genomewide reduction in DNA-binding proteins, notably histones. Thus, both mammalian *Hmgb1*−/− cells and *nhp6* yeast cells have fewer nucleosomes. This raised the critical question of where available nucleosomes are located when they are fewer. We found that, at least in yeast (but most likely also in mammalian cells), the reduction in nucleosome number does not alter nucleosome spacing and location, but reduces nucleosomal occupancy in a non-uniform way in different sites and is associated with an overall increase in transcript abundance and a specific alteration in the expression of a subset of genes.

## Results

### Mammalian Cells Lacking HMGB1 Are More Sensitive to Ionizing Radiation

Previous results indicated that a given dose of UV irradiation produced almost twice as many thymidine dimers in mammalian cells lacking HMGB1 and yeast cells lacking Nhp6 proteins compared to wild type cells [14]. We then asked whether ionizing radiation also produced more DNA damage in *Hmgb1*−/− cells.

**Figure 1:**
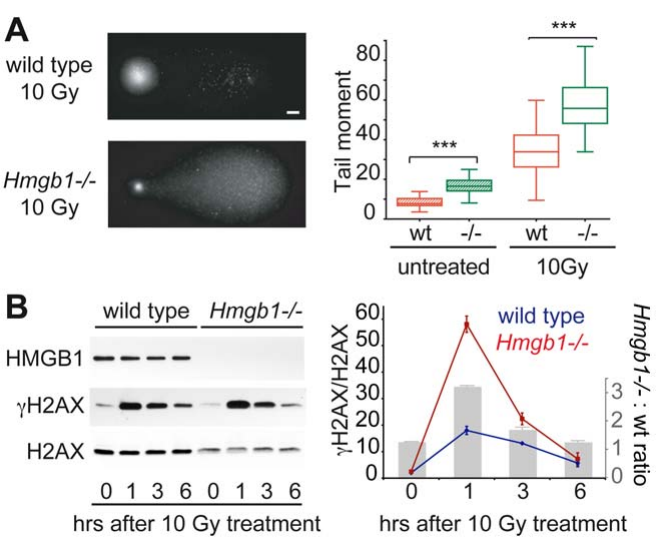
*Hmgb1*−/− nuclei are more accessible to DNA damage by ionizing radiation. (A) Left: visualization of DNA breaks by alkaline Comet Assay in G1-synchronised wild type and *Hmgb1*−/− cells after 10 Gy γ-ray irradiation. Bar: 10 µm. Right: quantification of DNA fragmentation before and after irradiation. Box plots: top and bottom mark the 25^th^ and 75^th^ percentiles; inner line, median; whiskers, maximum and minimum values. Tail moment values of wild type and *Hmgb1*−/− MEFs are statistically different both before and after irradiation (*p*<10^−4^, *n* = 50, *t* test). (B) Quantification of γH2AX in G1-synchronised wild type and *Hmgb1*−/− MEFs. Cells were irradiated as indicated in (A) and kept in culture for the indicated time before cell lysis. Western blotting for HMGB1, γH2AX, and H2AX were performed on equal amounts of total cell lysates. Data are expressed as γH2AX band intensities, normalized to total H2AX band intensities; error bars, SD of technical replicates in a representative experiment out of three performed. Quantifications were performed on images with different exposures within the linear part of the dynamic range. The histogram in gray shows the ratio of H2AX phosphorylation between *Hmgb1*−/− and wild type cells.

We irradiated primary wild type and *Hmgb1*−/− MEFs with 10 Gy of gamma rays; we measured the formation of single-stranded and double-stranded DNA breaks in individual cells by means of the comet assay [15], whereby in the presence of an electrophoretic field short DNA fragments migrate out of the lysed cell and into the agarose, whereas intact DNA remains confined (Figure 1A, left). The tail moment, which is a measure of DNA fragmentation, indicated that *Hmgb1*−/− MEFs contained more DNA breaks before irradiation (Figure 1A, right). The number of DNA breaks induced by irradiation was higher in *Hmgb1*−/− cells; this could not be ascribed to defective DNA repair since the cells were subjected to the assay immediately after irradiation, before DNA repair could deal with the breaks. We also quantitated γH2AX levels after irradiation with gamma rays (Figure 1B): substantially more H2AX is phosphorylated in *Hmgb1*−/− cells relative to wild type cells after 1 h, but the difference subsides after 6 h. This suggests that *Hmgb1*−/− cells can repair effectively double strand breaks.

Ionizing radiation generates hydroxyl radicals, which in turn react with DNA producing a large number of chemical modifications, including DNA breaks. Our results suggest that the DNA of *Hmgb1*−/− MEFs is more accessible to hydroxyl radicals.

### Cells Lacking HMGB1 Contain a Reduced Amount of Histones

DNA-bound proteins protect DNA from the attack of hydroxyl radicals; this property is exploited in protocols of hydroxyl radical footprinting. Nucleosomes shield DNA from hydroxyl radicals, and chromatin structure is a major factor determining DNA radiosensitivity [16]. We then hypothesized that the DNA of *Hmgb1*−/− cells is less protected by associated proteins, and in particular by histones. We thus measured histone content in *Hmgb1*−/− and wild type cells.

**Figure 2:**
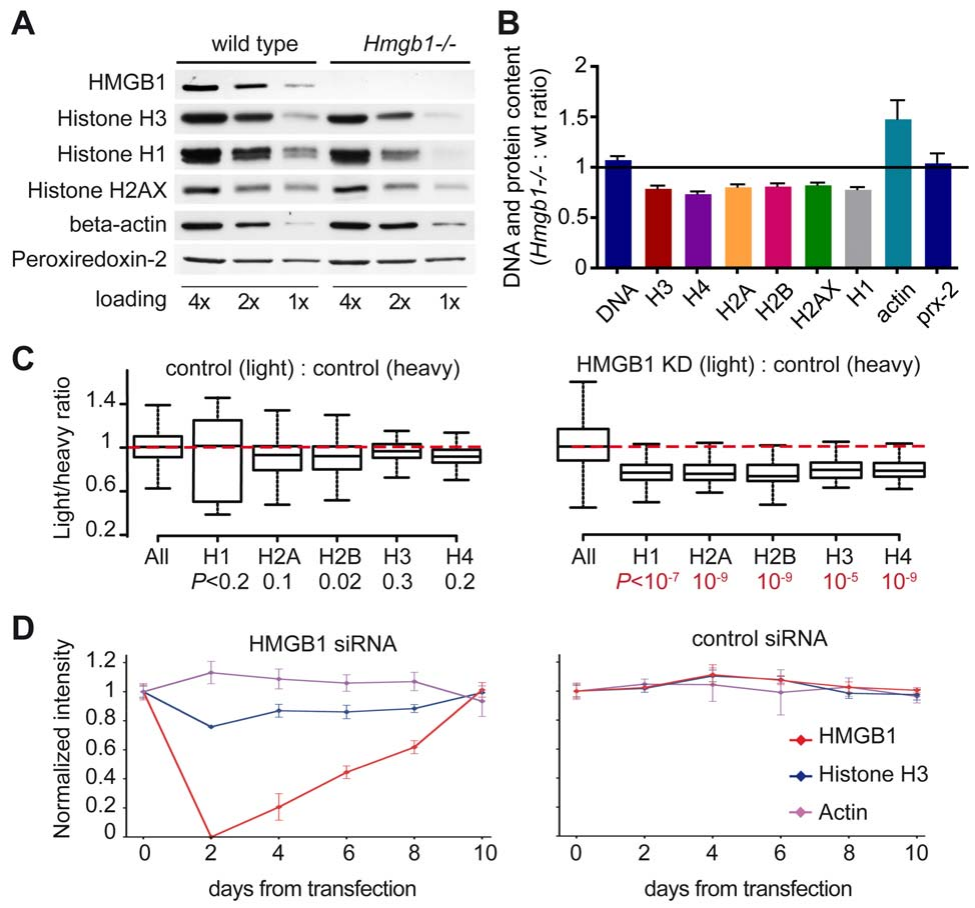
*Hmgb1*−/− cells contain a reduced amount of histones. (A) Western blot of serial 1∶2 dilutions starting from 50,000 G0/G1 synchronized cells. (B) Ratios of band intensities from the blots in (A) and two other similar experiments. Histone and actin ratios are significantly different from 1 (*p*<0.05, Wilcoxon test), while DNA and peroxiredoxin-2 ratios are not. Error bars represent SEM. (C) SILAC analysis of cellular histone contents. The box-plots represent Light/Heavy ratios for the whole proteome (“all,” all peptides) and non-modified peptides from histones. Left panel: control experiment where light- and heavy-labelled control cells were mixed (number of peptides: all peptides = 34,691, H1 = 39, H2A = 97, H2B = 70, H3 = 53, H4 = 147; mean values ± SD: H1 = 0.883±0.517, H2A = 0.882±0.168, H2B = 0.859±0.254, H3 = 0.942±0.162, H4 = 0.911±0.106). Right panel: experiment where light HeLa KD cells were mixed with heavy control cells (number of peptides: all peptides = 26,823, H1 = 42, H2A = 66, H2B = 48, H3 = 34, H4 = 81; mean values ± SD: H1 = 0.740±0.305, H2A = 0.769±0.185, H2B = 0.713±0.165, H3 = 0.763±0.116, H4 = 0.781±0.133). Probabilities are calculated using Wilcoxon test. (D) Quantification of the indicated proteins in HeLa cells transiently transfected with HMGB1 siRNA (left) or control firefly luciferase siRNA (right). Samples were collected at the indicated times after siRNA transfection and evaluated by western blotting. Error bars, SD from a representative experiment out of three performed.

We accumulated by Coulter counting an equal number of *Hmgb1*−/− and wild type MEFs, blocked in G0/G1 by serum starvation, and measured their DNA content by PicoGreen fluorescence and their histone content by quantitative immunoblotting (Figure 2A,B). While the amount of DNA was not statistically different between wild type and mutant cells, the amounts of core histones H2A, H2B, H3, and H4, linker histone H1, and the variant histone H2AX were all reduced by about 20% in *Hmgb1*−/− MEFs. On the contrary, beta-actin content (a common control for protein loading) was about 50% higher in *Hmgb1*−/− MEFs, whose cytoplasm appears larger than that of wild type MEFs even at an early passage (unpublished data). The abundance of other proteins, like peroxiredoxin-2, did not change in *Hmgb1*−/− MEFs.

We confirmed these results in HeLa cells stably transfected with a plasmid expressing HMGB1 shRNA (HeLa knockdown, henceforth KD) or a control plasmid. HMGB1 expression was almost abolished by the HMGB1 shRNA (Figure S1A), and cycling KD cells (Figure S1B) contained less core and linker histones (about 80% compared to the control HeLa cells) and more beta-actin (about 120%) (Figure S1C, upper panel).

We then compared the entire proteomes of control and KD cells by stable isotope labeling with amino acids in cell culture (SILAC) [17]. Control cells were grown for 8 passages in either light medium (Arg0 Lys0) or medium containing C13,N15-labelled arginine and lysine (Arg10 Lys8); KD cells were grown in light medium only. Light and heavy cells were mixed 1∶1 before lysis, subjected to SDS-PAGE and in-gel trypsin digestion; peptides were quantitated by liquid chromatography coupled to tandem mass spectrometry (LC-MS/MS) (Figure S2A). HMGB1-derived tryptic peptides were absent in KD cells, as expected (Figure S2B). In the control experiment (which compared heavy and light HMGB1-containing control cells) the ratios of light to heavy proteins had a narrow log-normal distribution (standard deviation close to 0.13). In contrast, when comparing light KD and heavy control cells these ratios showed a much wider distribution (standard deviation close to 0.37) (Figure S2C). The abundance of most proteins changed slightly but significantly, albeit few proteins showed a change larger than 2-fold (MS tracings for a few representative peptides are shown, Figure S2B). We then investigated the relative abundance of all histone-derived peptides that do not bear post-translational modifications; we excluded peptides known to bear modifications because a difference in their abundance could be due to variations in modification, rather than to a difference in the quantity of the histone protein. Notably, peptides from core and linker histones were reduced by about 25% in HMGB1-depleted cells (*p*<10^−5^ by two-sample Wilcoxon test) (Figures 2C, S2D). Variant histones H2AX and H2AZ were also significantly reduced (Figure S3). These experiments were repeated on wild type and *Hmgb1*−/− MEFs, with comparable results (unpublished data).

Taken together, SILAC and quantitative immunoblotting indicated that cells lacking HMGB1 contain a coordinately reduced amount of all histones.

### The Histone Content of Mammalian Cells Can Be Reduced Transiently and Reversibly

A lower histone content might be due to compensatory mutations selected in response to the lack of HMGB1 in *Hmgb1*−/− cells. In this case, rare mutant cells might be selected during in vitro culture and expand into viable clones. Alternatively, all cells might be able to modulate histone content in response to their physiological state, including the available level of HMGB1 protein. To distinguish between the possibilities, we transfected HeLa cells with 21-mer double-stranded HMGB1 siRNA, verified the disappearance of HMGB1, and grew the cells for 10 d until the amount of HMGB1 returned to normal (Figure 2D). Notably, the amount of histone H3 decreased concomitantly with the decrease in HMGB1, down to less than 80% of the starting level, and then recovered concomitantly with the recovery in HMGB1 content. HeLa cells transfected with control firefly luciferase siRNA showed no change in either HMGB1 or histone H3 content. Since there was no gross cell death after siRNA transfection, cells with a reduced histone content are not rare clones selected from a large cell population; rather, most cells in the population down-regulate histone content in response to a lack of HMGB1, and this regulation is reversible.

To further establish the physiological interdependence between HMGB1 and histone content we examined MEFs derived from *Hmgb1*+/− heterozygous embryos; these have one half the amount of HMGB1 protein and contain about 90% of the normal amount of histones, which is intermediate between the amount in wild type and in *Hmgb1*−/− MEFs (unpublished data). Finally, we verified that *Hmgb1*−/− embryo livers contain a 20% reduction in histone content (Figure S4), further excluding that the observed histone reduction in cultured cells can be due to culture conditions.

### Cells Lacking HMGB1 Contain Fewer Nucleosomes

**Figure 3:**
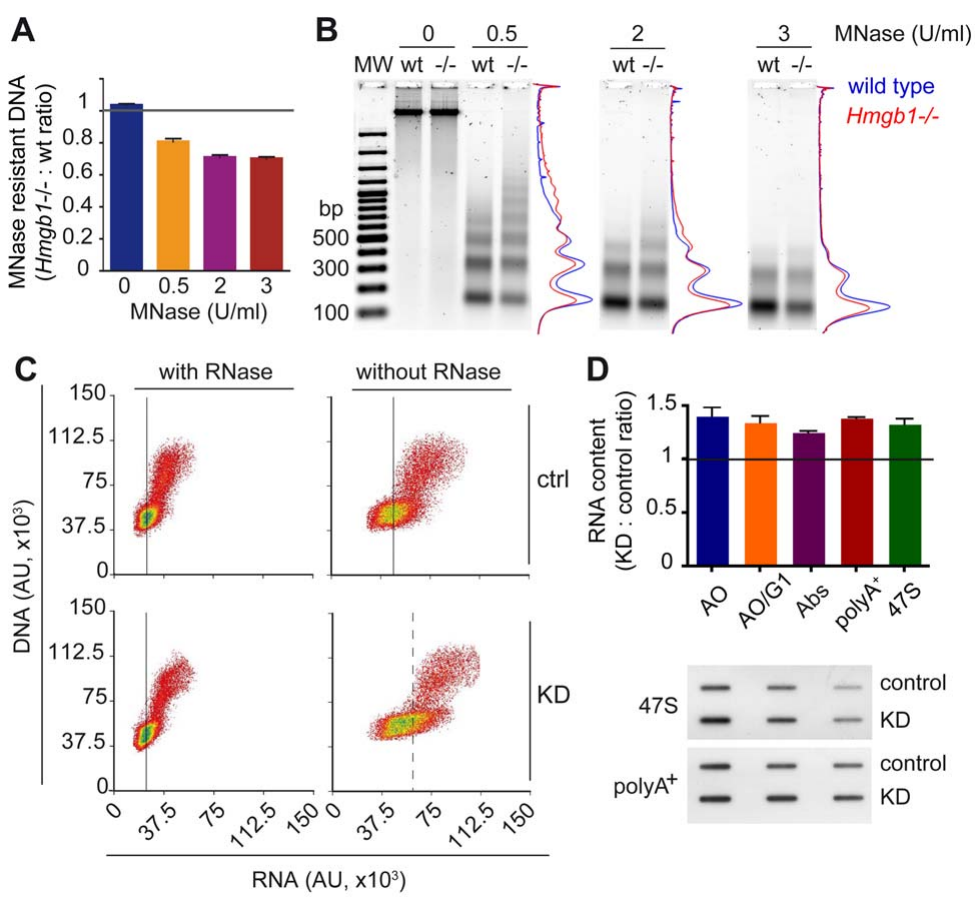
Cells lacking HMGB1 contain fewer nucleosomes and more RNA transcripts. (A) Residual (nucleosome-protected) DNA obtained from *Hmgb1*−/− and wild type MEFs after digestion with increasing MNase concentrations. Error bars, SEM from three biological replicates. (B) Electrophoretic separation and densitometric analysis of DNA samples from 250,000 wild type and *Hmgb1*−/− MEFs after digestion with 0, 0.5, and 2 U/ml of MNase. MW: 100 bp ladder. (C) FACS analysis of HeLa control (upper panels) and KD cells (lower panels) stained with Acridine Orange (AO), with or without prior RNase treatment (left and right, respectively). Fluorescence from AO bound to DNA (*y*-axis, 530/30 nm) and to RNA (*x*-axis, 610/20 nm). Black vertical lines (continuous and dashed) indicate the arithmetic means of RNA fluorescence in G1 cells. (D) Quantification of total RNA content in control and KD HeLa cells by FACS (cycling and G1) and by 260 nm absorbance of RNA extracted from a defined number of cells. Quantification of polyA^+^ mRNA and 47S rRNA precursor by RNA slot blot hybridization with specific probes (lower panel, details in Material and Methods). Error bars, SEM of three biological replicates. RNA ratios are significantly different from 1 (*p*<0.05, Wilcoxon test).

Histones are predominantly associated with DNA to form nucleosomes. Thus, a severe reduction in histone content should translate in a corresponding reduction in nucleosomally organized DNA. To verify the hypothesis that the DNA of *Hmgb1*−/− cells might be wrapped into fewer nucleosomes, cells were partially lysed and chromatin was digested with increasing amounts of micrococcal nuclease (MNase). At higher MNase concentrations, the amount of remaining (nucleosome-protected) DNA was reduced by about 30% in *Hmgb1*−/− MEFs (Figure 3A, quantification with PicoGreen). Agarose electrophoresis indicated that the total amount of MNase-resistant DNA is reduced in *Hmgb1*−/− MEFs, at all concentrations of MNase (Figure 3B). However, at low MNase concentration (0.5 U/ml, Figure 3B), *Hmgb1*−/− samples contained more of higher molecular weight DNA (Figure 3B). This result was repeated several times and most likely indicates that a minor fraction of the chromatin of *Hmgb1*−/− cells becomes more resistant to digestion, in contrast to the major fraction which becomes more accessible.

The average spacing between nucleosomes was very similar in *Hmgb1*−/− and wild type MEFs (Figure 3B), contrary to what is expected if available nucleosomes were uniformly redistributed over the genome. Similar results were obtained with KD cells (Figure S1C,D).

The conclusion from these experiments is that mammalian cells can survive and proliferate with substantially fewer nucleosomes.

### Cells with Fewer Nucleosomes Contain More RNA Transcripts

The availability of cells with fewer nucleosomes allowed us to test the widely held opinion that nucleosomes limit transcription in vivo, as they do in vitro by impeding the progress of RNA polymerases [18],[19]. We quantified total nucleic acids in KD and control HeLa cells by FACS after acridine orange staining (Figure 3C) [20]. Whereas the DNA content was similar in KD and control cells, the RNA content is about 1.3 times higher in KD cells. Both polyA^+^ mRNA and the 47S rRNA precursor are similarly increased (Figure 3D).

Although global transcript abundance increases in cells lacking HMGB1, so that most transcripts will be more abundant, the expression of individual genes can also change relative to each other. We thus measured the relative representation of each transcript within an identical amount of RNA extracted from cells. Relative representation in a fixed amount of RNA automatically normalizes away the global increase of about 30% in total RNA amount in HeLa KD cells. The comparison in relative amount is instructive to identify which genes deviate the most from the average effect. Our analysis indicates that about 13% of transcripts (1,080 over 8,027 on the Illumina platform; *p*<0.01; Figure S5A) are over-represented (577 genes) or under-represented (503 genes) from the average 30% increase in KD HeLa cells. The Gene Ontology categories significantly affected at the mRNA level (*p*<0.05, Wilcoxon test) are indicated in Figure S6A. These broadly correspond to the Gene Ontology categories significantly affected at the protein level (Figure S6B).

### HMGB1 Promotes Histone Deposition In Vitro

Since the absence of HMGB1 leads to a decrease in nucleosome number, we investigated whether HMGB1 was directly involved in chromatin assembly, as suggested by early experiments [21].

**Figure 4:**
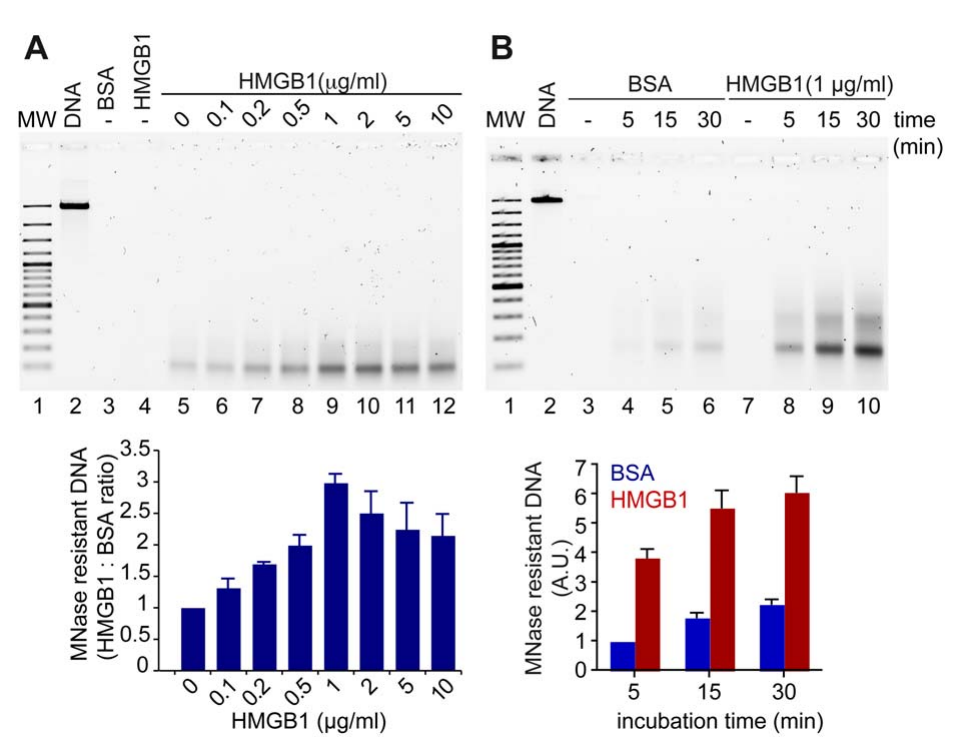
HMGB1 promotes the assembly of chromatin in vitro. (A) Chromatin was assembled in vitro on linear DNA using purified histones, hNAP1, ACF, and increasing amounts of HMGB1 protein; then it was digested with MNase. The residual DNA after digestion was electrophoresed on a 1.5% agarose gel (upper panel) and quantified with PicoGreen (normalized to the reaction containing BSA, lower panel). Error bars, SD of three replicates. (B) Chromatin was assembled in the presence of a fixed amount of HMGB1 (1 µg/ml), or BSA as control, for the indicated time points. Electrophoresis (upper panel) and quantification by PicoGreen (lower panel) of the residual DNA after digestion with MNase are shown. Error bars, SD of three replicates. MW: 100 bp ladder.

We tested the effect of HMGB1 on histone deposition onto naked DNA using a simple, commercially available assay (Chromatin Assembly Kit by Active Motif). Linearized plasmid DNA was mixed with soluble histones, the histone chaperone NAP, and the remodeling factor ACF. After incubation for 15 min at 27°C, the assembled chromatin was digested with micrococcal nuclease, and an aliquot was run on an agarose gel (Figure 4A, upper panel). No DNA remained after nuclease digestion if histones were absent from the assembly reaction (lane 3–4), whereas a clear band of mononucleosomal size was present in the presence of histones (lane 5). We then added to the reaction mix increasing concentrations of HMGB1, and we noted a highly significant dose-dependent increase in the mononucleosome band (lanes 6–9), reaching a maximal yield at 1 µg/ml. At higher HMGB1 concentrations, the efficiency of nucleosome deposition decreased (lanes 10–12). At the optimal HMGB1 concentration, nucleosome formation was 3.5 times faster in the presence than in the absence of HMGB1 (Figure 4B, upper panel). Direct quantification of nuclease-resistant DNA by PicoGreen confirmed the data obtained by gel electrophoresis (Figure 4A and B, lower panels).

### *nhp6* Yeast Mutants Recapitulate the Phenotype of Mammalian Cells Lacking HMGB1

**Figure 5:**
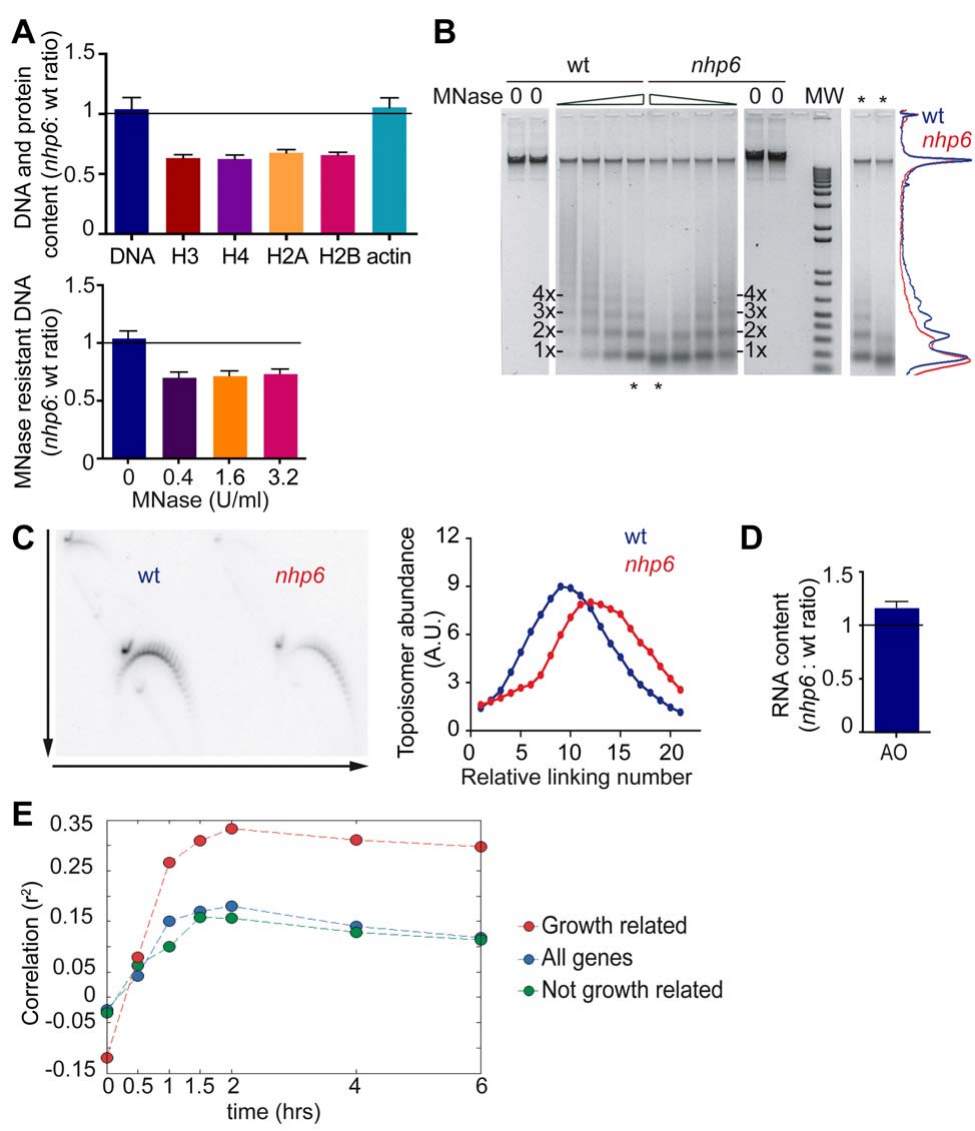
*nhp6* cells contain fewer nucleosomes and more RNA transcripts. (A) Quantification of histone content (upper panel) from western blots of wild type and *nhp6* cells; the decreases in core histone contents are statistically significant (*p*<0.05, Wilcoxon test). Lower panel: residual (nucleosome-protected) DNA obtained from *nhp6* and wild type cells after digestion with increasing MNase concentrations. Error bars represent SEM from three biological replicates. (B) Electrophoretic separation of DNA samples from 3×10^8^ wild type and *nhp6* cells after MNase digestion (from 6.4 U/ml in 2× dilutions). The densitometric analysis of the central two lanes (asterisks) is shown on the right. (C) Topological analysis of yRp17 plasmid in wild type and *nhp6* cells by 2D-electrophoresis in the presence of different amounts of chloroquine in orthogonal directions (arrows in the left panel). Quantification of the different DNA topoisomers is shown in the right panel. (D) RNA quantification in wild type and *nhp6* cells by Acridine Orange staining. Error bars, SEM of three biological replicates. RNA ratio is significantly different from 1 (*p*<0.05 Wilcoxon test). (E) Correlation over time between gene expression profiles of UKY403 and *nhp6* cells. Time 0 corresponds to the galactose to glucose shift for the UKY403 strain.

Yeast Nhp6 proteins are functionally equivalent to HMGB1 in mammalian cells, and *nhp6* yeast mutants are more sensitive to UV irradiation [14]. We therefore verified whether yeast *nhp6* cells also have reduced histone and nucleosome content. We synchronized yeast cells in G1 by treatment with alpha factor pheromone, collected an equal number of wild type and *nhp6* cells, and measured their DNA content with PicoGreen and their histone content by quantitative immunoblotting. *nhp6* cells contained about 65% of the amount of histones compared to the wild type, and their chromatin was more accessible to digestion by MNase (Figure 5A,B). Moreover, 2D gel analysis indicated that the supercoiling of the 7.0 kb yRp17 plasmid was reduced by about three turns in *nhp6* cells, equivalent to about three nucleosomes fewer than in the wild type (Figure 5C). Finally, *nhp6* cells contain about 1.2 times more RNA than wild type cells (Figure 5D). We conclude that the phenotypes of *nhp6* and *Hmgb1* mutants are largely similar.

### Yeast Cells with a Reduced Nucleosome Number Have a Distinct Transcriptional Profile

A transient model of nucleosome depletion in yeast was examined previously [22]. In the UKY403 yeast strain, the sole copy of histone H4 is under the control of the *GAL1* promoter. In glucose medium, UKY403 cells lost around 50% of nucleosomes by 6 h, relative to a control strain with a wild type H4 gene, and the expression of 15% of genes increased and the expression of 10% of genes decreased more than 3-fold.

We then looked at the relative expression of genes in wild type and *nhp6* cells and compared them to those in the UKY403 strain. By Affymetrix analysis we found that out of 5,447 genes, 219 are up and 251 are down in *nhp6* relative to wild type cells (1.5-fold threshold and *p*<0.05) (Figure S5B). The Gene Ontology categories that are significantly affected are shown in Figure S7. The correlation between gene expression profiles in UKY403 and *nhp6* cells (Figure 5E) rises from r^2^<0, when nucleosomes are not depleted in UKY403, to almost 0.16 after 2 h (*p*<10^−33^), and remains almost constant thereafter. Since about half of the genes transcriptionally affected by nucleosome depletion in the UKY403 strain are also affected by slow growth, we asked whether our results were influenced by the slow growth of the *nhp6* mutant relative to its wild type counterpart [13]. Indeed, the correlation between the two strains is much stronger for the growth-related gene subset; however, the correlation for the genes unresponsive to changes in growth rate is only slightly smaller (r^2^ = 0.15, *p*<10^−5^) than the one for all genes. Taken together, these data suggest that nucleosomal depletion affects transcription profiles in broadly similar ways in strains where histone H4 is depleted or Nhp6a/b proteins are lacking.

### In Yeast Cells a Reduced Nucleosome Number Affects Primarily Nucleosome Occupancy and Not Nucleosome Position

Research in the last few years has highlighted the importance of nucleosome positioning in the control of transcription; we therefore asked how nucleosome depletion affects genomewide nucleosome positioning.

**Figure 6:**
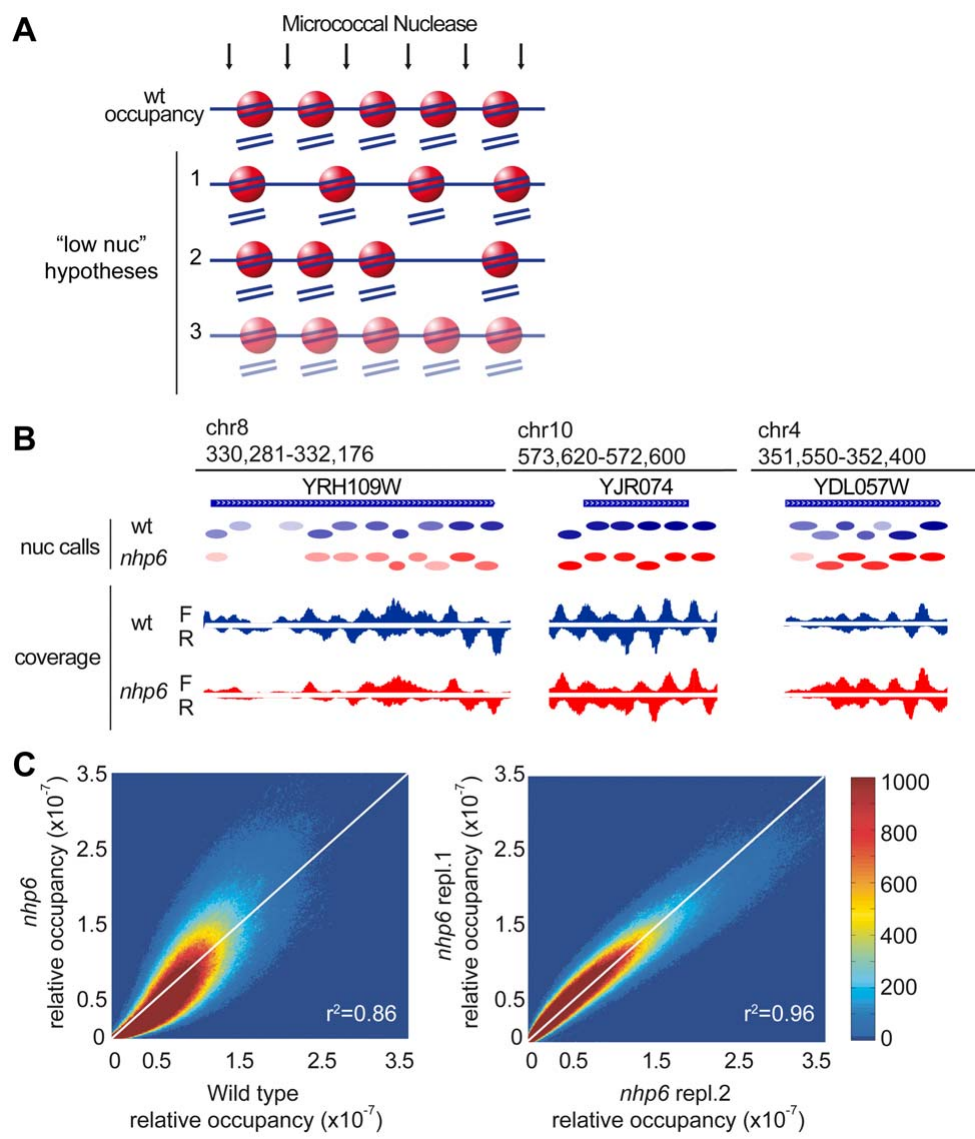
*nhp6* cells have increased variability in nucleosome occupancy. (A) Schematic diagrams representing possible distributions of nucleosomes in low-nucleosome conditions. Nucleosomes are depicted as red spheres, DNA as a blue line. In wild type cells five nucleosomes cover ∼1 kb of DNA (first row). The schemes represent a 20% reduction in nucleosome content: 4 nucleosomes can redistribute over 1 kb (hypothesis 1), or 1 nucleosomal site is left vacant (hypothesis 2), or all 5 sites are occupied by nucleosomes but only 80% of the time relative to the wild type (hypothesis 3). (B) High throughput sequencing of MNase-resistant DNA shown for three loci of the yeast genome; wild type (blue) and *nhp6* cells (red). Ovals represent nucleosomes called by template filtering; colour saturation is proportional to relative occupancy. F and R: Forward (sense) and Reverse (anti-sense) reads. (C) Density dot plots showing the relative occupancy per bp. Left: wild type (*x*-axis) versus *nhp6* cells (*y*-axis); right: two biological replicates of *nhp6* cells. The colour of each point represents the number of base pairs that map to that point in the plot. Pearson correlation coefficients are shown in the right bottom corner of the plots.

The hypothesis of statistical positioning states that nucleosomes space themselves between barriers [23]. In this case, the position of nucleosomes should vary when their number is lower (Figure 6A, hypothesis 1). According to the alternative hypothesis that DNA sequence is the major determinant of nucleosome positioning, nucleosome limitation could lead to the selective loss of a minority of nucleosomes (hypothesis 2). Alternatively, nucleosomes might occupy the same positions, but spend less time on each of them (hypothesis 3; nucleosome “occupancy” of individual sequences is reduced).

When we applied high throughput sequencing to MNase-resistant DNA from *nhp6* and wild type cells, we found that the distribution of sequence reads was very similar. Representative snapshots of the nucleosome maps are shown in Figure 6B; a complete browsable form is available at the website indicated in Materials and Methods.

The number of times a specific base pair appears in sequence reads, divided by the total number of sequence reads, is the relative occupancy of that base pair. Relative occupancies of all base pairs can then be compared between strains; a density dot plot allows a visual representation of such a comparison. The comparison between biological replicates gives a density plot where most bases cluster around the diagonal (Figure 6C, right). The comparison between *nhp6* and wild type cells (Figure 6C, left) gives a density plot which is more dispersed about the diagonal and has a characteristic skew with more points below the diagonal at low occupancy and more points above the diagonal at high occupancy. This result is inconsistent with a global redistribution of nucleosomes over the genome (hypothesis 1 in Figure 6A), which would give a smeared density plot with a lot of points close to the axes (base pairs occupied in one strain but not in the other). Our result is also inconsistent with the disappearance of nucleosomes from a minority of sites (hypothesis 2), which would give a density plot with two separate sub-populations, as simulated in Figure S8A.

In fact, the similarity of the *nhp6*/wt density plot to that of biological replicates indicates that most base pairs that are occupied by nucleosomes in wild type cells are also occupied in *nhp6* cells. A complete identity between nucleosome positions in the two strains (although with reduced occupancy in one strain) would give the same density plot of biological replicates. Thus all three hypotheses depicted schematically in Figure 6A do not correspond to observation, but hypothesis 3 comes closest.

We next moved from coverage at individual base pairs to examination of nucleosome positions. We used template filtering [24] to call nucleosome positions and confirmed that they are highly conserved (Figure 6B, “nuc calls”). Almost half of the nucleosomes are centered around the same position in both strains, many are offset by about 10 base pairs and some by 20 pairs (Figure S8B); we note that 10-bp shifts correspond to those expected from the rotational periodicity of DNA wrapped around the nucleosome. Only about 30% of nucleosomes had shifted by more than 20 base pairs. This confirms that most nucleosomes occupy approximately the same location in the two strains. However, in *nhp6* cells fewer nucleosomes were unambiguously called (45,441 versus 53,643), and the read peaks that identify nucleosome edges were broadened in the *nhp6* sequencing data (Figure 6B). This suggests that some nucleosomes may shift from a single favored position into a superposition of multiple overlapping positions (“fuzzy nucleosomes”; [23]); beyond a certain degree of fuzziness, nucleosomes would not be called by the algorithm. The length of DNA predicted to be covered by nucleosomes was reduced on average and had increased variability in *nhp6* cells (Figure S8C), consistent with increased fuzziness.

As observed at the single base pair level (Figure 6C), many nucleosomal sites are either less or more relatively occupied in *nhp6* cells. This is clearly visible in the snapshots in Figure 6B, showing three different loci with decreased, unchanged, and increased relative occupancy, respectively. Absolute occupancy is proportional to the nucleosome number, and thus is reduced by about 30% in *nhp6* cells. As a result, on some sites absolute occupancy in *nhp6* cells may be comparable to that in the wild type (but not higher), whereas on the vast majority of sites it will be reduced or very reduced. High-resolution primer extension analysis confirmed a similar position of nucleosomes in the *ars1* locus, but with higher accessibility of nucleosome-covered sequences (and thus lower absolute occupancy) in *nhp6* cells (Figure S9A). Nucleosome ChIP (using an antibody against histone H3) also was in agreement with reduced nucleosome occupancy of the *ars1* locus in *nhp6* cells (Figure S9B).

Overall, these results are in accordance with increased chromatin accessibility in the *nhp6* mutant and suggest that nucleosomes have increased mobility on the sites they occupy (either intrinsic or catalyzed by nucleosome remodelling complexes).

### Nucleosome Position and Occupancy Over Gene Control Regions in *nhp6* Cells

**Figure 7:**
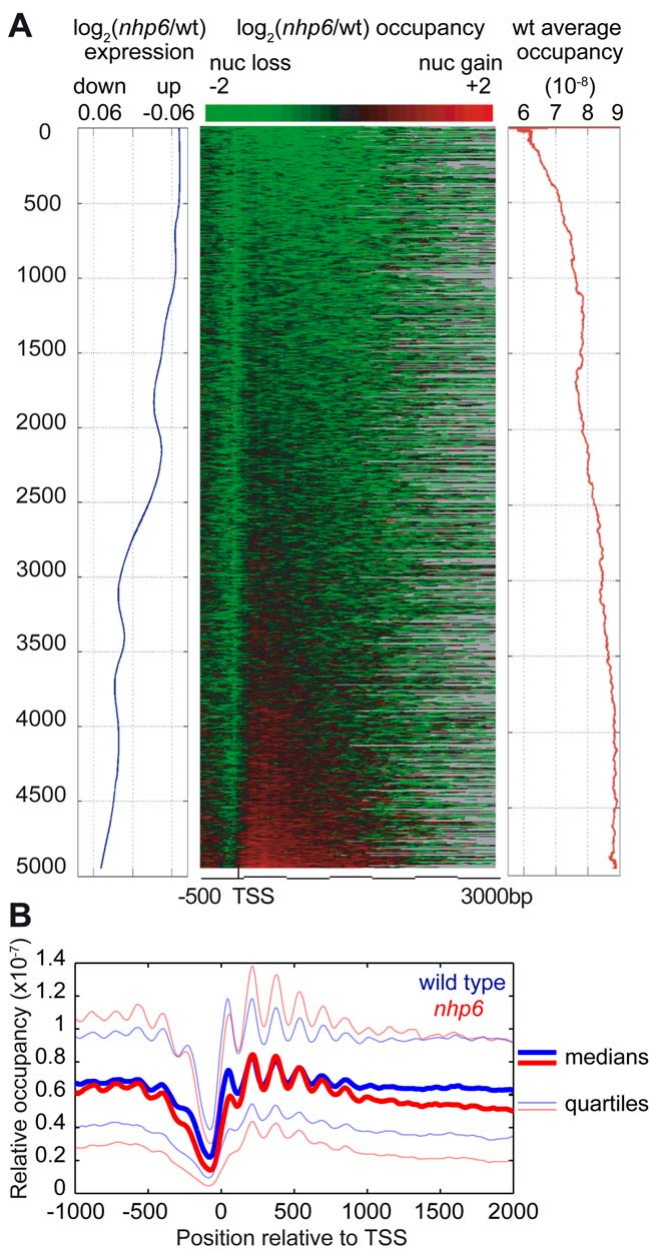
Nucleosomal occupancy on yeast coding genes. (A) Correlation between nucleosomal occupancy over genes and mRNA levels. Left, smoothed moving average of the expression fold changes for 4,945 genes. Center, genes aligned by their TSS were sorted by the log_2_ *nhp6*/wild type ratio of nucleosome occupancy. Gray is used for genes whose CDS is shorter than 3 kb. Right, wild type relative nucleosomal occupancy averaged over the entire gene. (B) Nucleosome coverage over 4,945 genes aligned by TSS. Thick blue and red lines indicate the median occupancy for wild type and *nhp6* cells, respectively; the 0.25 and 0.75 quartiles are shown as blue and red thin lines.

Nucleosomal organization of the control regions of genes is considered most important for gene expression. In yeast, nucleosomes are regularly arranged on protein-coding genes, starting from the transcriptional start site (TSS). A nucleosome-depleted region (NDR, also called nucleosome-free region, NFR) of about 140 bp is generally found just upstream of the TSS and is surrounded by two well-positioned nucleosomes, called −1 and +1 nucleosome, respectively. We aligned genes by their TSS and ranked them by the severity of nucleosome loss in *nhp6* cells relative to wild type (Figure 7A, heatmap in the center). All genes had reduced occupancy of the −1 nucleosomes (green streak in the heatmap), and genes with more severe nucleosome loss at the 5′ end also had reduced nucleosome occupancy over the gene body. Once again, we observed that the genes with more severe nucleosome loss in *nhp6* cells (Figure 7A, center) were the ones already low in nucleosome occupancy in the wt (Figure 7A, right, red line). A few genes appeared to have relatively increased occupancy in *nhp6* cells (red genes in the bottom of Figure 7A); these genes belong primarily to the Gene Ontology categories “metabolism” and “cell wall”. None of these genes, however, appeared to have increased absolute nucleosome occupancy (i.e., after considering that nucleosome number is reduced by about 30% in *nhp6* cells).

Nucleosome by nucleosome, median relative occupancy over the promoter and the TSS of all genes (from the −1 to the +1 nucleosome) was lower in *nhp6* cells, and median relative occupancy for the +2, +3, and +4 nucleosomes was slightly higher (thick lines in Figure 7B). Relative occupancy of all nucleosomal sites is more variable in *nhp6* cells (the thin lines in Figure 7B indicate the lower and upper quartiles of occupancy). Lower nucleosome occupancy in the control regions correlates with increased gene expression (Figure 7A, left, blue line, and Figure S8D).

### A Simple Model Predicts Nucleosome Occupancy in *nhp6* Cells Starting from Nucleosome Occupancy in Wild Type Cells

In all our analyses, from the correlation of base pair occupancy to the distribution of nucleosomes over genes, a theme stands out: the reduction in nucleosome number is associated with an increase in the variability of relative occupancy. From the point of absolute occupancy, we have already pointed out that some sites might be similarly occupied in wild type and *nhp6* strains, while sites that are intermediately occupied in the wild type are less occupied in *nhp6* cells, and weakly occupied sites in the wild type are much less occupied in *nhp6* cells. The skew in the density plot of Figure 6C visually represents this pattern of more pronounced loss of occupancy in *nhp6* cells from sites that are already less occupied in the wild type.

**Figure 8:**
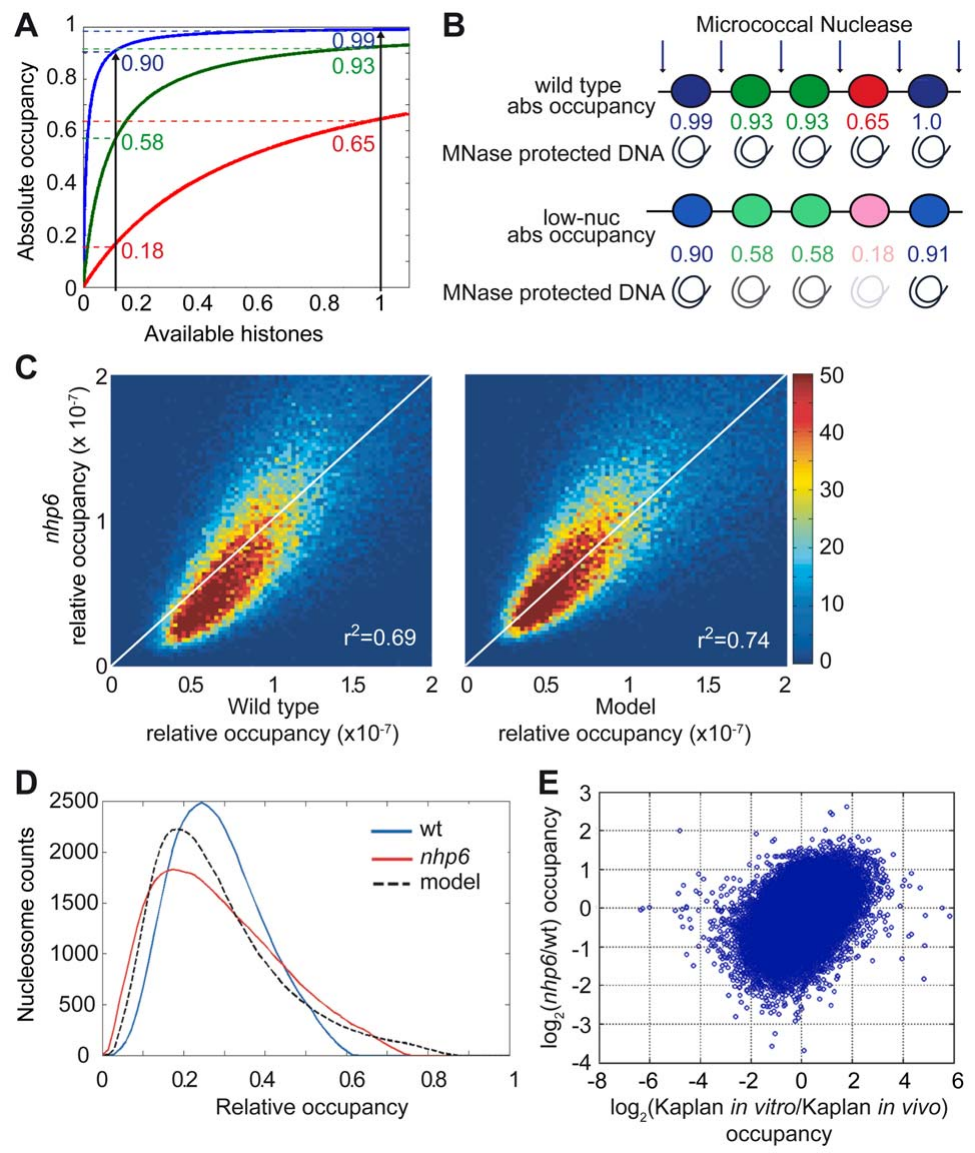
Mathematical model describing nucleosomal occupancy. (A) Affinity model based on saturation of nucleosomal sites. Since there are more DNA sites that can be assembled into nucleosomes than histone octamers, the occupancy of each site (*y*-axis) will be determined by the availability of non-nucleosomal histones (*x*-axis) and the relative dissociation constant (k_i_) of histones at that site (blue, low k_i_; green, medium k_i_; red, high k_i_). Upon decreasing the availability of histones (black vertical line on the left), the occupancy of sites with high dissociation constant decreases more than the occupancy of sites with low dissociation constant. This simple model resembles a Michaelis-Menten association, described by hyperbolic curves. (B) Absolute occupancies of hypothetical nucleosomal sites with high (blue), intermediate (green), and low (red) affinity in conditions of normal (wild type) or low nucleosome content. (C) Density dot plots showing relative occupancy of ∼50,000 nucleosomes in wild type (*x*-axis) versus *nhp6* cells (*y*-axis) (left panel) and observed *nhp6* nucleosome occupancy versus the occupancy predicted by the model (right panel). The colour of each point represents the number of nucleosomes that correspond to that point in the plot. Pearson correlation coefficients are shown in the right bottom corner of the plots. (D) Distribution of relative nucleosome occupancy measured in wild type (blue) and *nhp6* cells (red) and predicted distribution in *nhp6* cells by our model (black, dashed). (E) The scatterplot shows the comparison of changes in nucleosomal occupancy between our *nhp6*/wild type datasets (*y*-axis) and the in vitro/in vivo datasets from Kaplan et al. (*x*-axis). Each dot is a nucleosome as in (C), axes are in log_2_ scale. Correlation between the two pairs is r^2^ = 0.46 (*p*<10^−6^).

Unequal occupancy of nucleosomal sites in vivo is expected, since (1) in vitro the probability of nucleosome occupancy on different sites can vary by a factor of up to 5,000 [25] and (2) histone octamers are insufficient to package all the genome [26]. Thus, in physiological conditions some sites will be occupied close to 100% of the time (“saturated”) and some much less. Based on these considerations, we designed a model to account for the characteristic pattern of nucleosomal occupancy in *nhp6* cells. We assume that all sites compete for a finite pool of histones that is insufficient for all of them, and that each site has a certain probability of being occupied, that depends from histone availability. We also posit that the probability of occupation versus availability of histones is a hyperbolic function and is different for each site (Figure 8A). This model recalls formally the formation of a complex between two macromolecules, and we can thus assign a dissociation constant k_i_ to each nucleosome. The occupancy O of site i is then O_i_ = *x*/(*x*+k_i_), where k_i_ is the dissociation constant and *x* is the concentration of available histones. A decrease in the availability of histones will result in a skewed desaturation, with heavy nucleosome loss at sites of high dissociation constant and mild loss at sites of low dissociation constant (Figure 8A,B). This will increase the variability of relative occupancy.

Based on the relative occupancy of wild type sites, the model should be able to predict the genomewide occupancy for a certain decrease in available histones (details in Figure S8E). We then used our model to simulate the relative occupancies in a population of cells which have a 30% reduction in histone content (Figure 5A). The density dot plot comparing *simulated* and *observed* occupancy in *nhp6* cells (Figure 8C, right) is almost symmetrical about the diagonal and corrects the observed systematic skew in the density dot plot of *nph6*/wt relative occupancies (Figure 8C, left), although the dispersion of values is not decreased substantially. We also plotted the distribution of the number of nucleosomes at each occupancy value (Figure 8D); our model correctly predicts the approximate shape of the distribution for *nhp6* cells and the position of the mode. The fitting between the observed and predicted *nhp6* occupancies is optimal at the nucleosome content actually observed for *nhp6* cells. An alternative model based on statistical positioning does not justify our observations, since it predicts that both position and spacing of nucleosomes would be changed when histone content is reduced (Figure S8F, red line), contrary to what we observed.

Overall, our model justifies the disproportionate loss of nucleosomes from weakly occupied sites, and the increase in relative occupancy at the more occupied sites (Figure 8D, far tail of the distribution). We then asked whether the sites with reduced occupancy in *nhp6* cells are the ones with lower intrinsic ability to form nucleosomes. To this aim, we compared our dataset with the dataset obtained by reconstituting yeast chromatin in vitro [27]. The comparison of changes in nucleosomal occupancy between our *nhp6*/wt dataset and Kaplan's in vitro/in vivo dataset is shown in Figure 8E. The Pearson correlation coefficient between datasets is r^2^ = 0.46 (*p*<10^−6^), indicating that the sites that most lose occupancy in *nhp6* cells correspond to the ones with lower occupancy in reconstituted chromatin; conversely, the sites that lose less occupancy in *nhp6* cells correspond to the ones that most easily reform chromatin in vitro (Figure 8E).

## Discussion

### Nucleosome Number Is Not Fixed

We show here that the absence of HMGB1 or Nhp6a/b proteins causes substantial histone and nucleosome depletion in mammalian and yeast cells; surprisingly, nucleosome depletion is compatible with cell survival.

While our work was in progress, a substantial reduction in the histone content in chromatin was described in aging yeast cells [5]; forced expression of plasmid-borne histone genes was shown to increase the lifespan of yeast cells. Our results independently confirm that yeast cells can contain a variable amount of chromatinized histones and that reduced histone content leads to aging: *nhp6* yeast mutants, which we show have a reduced histone and nucleosome content, have a reduced lifespan and an increased level of extrachromosomal ribosomal DNA circles, which are a hallmark of aging [14]. Histone depletion has also been demonstrated in aging mammalian cells [6].

We also confirm a correlation between nucleosome depletion and an increase in DNA damage [6]. We suggest that a decrease in nucleosome number increases DNA damage because DNA is more exposed to DNA damaging agents, as indicated by in vitro experiments showing that nucleosomes protect DNA from hydroxyl radicals [16]. Reactive oxygen species, including hydroxyl radicals, are produced also by basal metabolism [28],[29], which may explain an increase in DNA breaks in non-irradiated *Hmgb1*−/− cells.

We also report that nucleosome depletion correlates with a global increase in transcript abundance. Our observation in living cells supports the current notion (based on experiments in vitro) that nucleosomes limit the accessibility of DNA to the transcription machinery.

We have asked what happens to the basic organization of eukaryotic genomes when only a limited number of nucleosomes can be formed. The prevailing view was that histones are deposited until all DNA is packaged. Indeed, Kornberg and Stryer [30] proposed that the fairly uniform spacing of nucleosomes along DNA arises from the “statistical positioning” of nucleosomes between fixed barriers, so that when the DNA is saturated with histones, each nucleosome finds itself within a narrow distribution of distances from the preceding nucleosome. As a consequence, when the number of nucleosomes is reduced, the distance between nucleosomes should increase. This is not what we find, neither in yeast nor in mammalian cells.

At least in yeast, nucleosomes largely occupy the same positions also when nucleosome number is lower than usual; nucleosome occupancy drops, but not uniformly. In general, sites that are highly occupied in wild type cells remain highly occupied also in nuclesosome-poor *nhp6* cells, whereas nucleosomes are mostly lost from sites that already had low occupancy in wild type. This creates a ranking of sites for nucleosome occupancy, which can be at least partially explained by a model that assumes formally that each site has its own affinity for histones available for deposition. This is certainly compatible with models that affirm that nucleosome position is driven largely by DNA sequence. Indeed, the comparison between Kaplan's and our datasets indicates that the sites that are more nucleosome-depleted in *nhp6* cells are the ones with lower intrinsic propensity to form nucleosomes. Anyway, our model does not exclude strong contributions to nucleosomal location by active processes such as nucleosome deposition, sliding, eviction, or remodeling, as will be discussed in the following sections.

### Critical Issues in Determining Nucleosome Location and Occupancy

Nucleosomal position is measured directly in yeast, by identifying the borders of MNase-resistant DNA sequences, as is relative nucleosomal occupancy, by dividing the number of reads of a specific MNase-resistant sequence by the total number of MNase-resistant sequence reads. However, absolute nucleosomal occupancy is calculated by multiplying relative occupancy by the amount of nucleosomally organized DNA. We consider that DNA remaining after MNase digestion represents nucleosomally organized DNA, but an alternative explanation is often favored: chromatin may become more accessible to MNase not because of a variation in nucleosome number but because of unspecified changes in higher-order chromatin organization. This alternative explanation is not compatible with the topological analysis of plasmid supercoiling (Figure 5C), which does not depend on MNase digestion nor in fact on any other type of chromatin accessibility, but only on the number of nucleosomes residing on the plasmid at the time of extraction. Most of all, the alternative explanation does not fit with the reduced abundance of histones: since only about 0.1% of histones are not engaged within nucleosomes [31], a reduced amount of histones must be reflected in a decreased number of nucleosomes.

Measuring histone content is therefore critical in interpreting alterations in chromatin organization. Nucleosomes were also recently mapped in *nhp6* cells by Dowell et al. [32]; our results, despite the different approach (high throughput sequencing versus hybridization to tiled chips), broadly agree with theirs, but the interpretations are different. Dowell et al. infer that Nhp6 proteins stabilize nucleosomes directly, possibly by interacting with them; we infer that nucleosome occupancy is substantially reduced in most nucleosomal locations, due to decreased histone content. Although they are different, the two interpretations are not mutually exclusive, and indeed specific interaction with Nhp6 proteins might explain the preferential loss of nucleosome +1 at the 5′ end of genes (Figure 7A,B) [32].

Other yeast mutants (for example, *spt10*; [33]) show an “altered” organization of chromatin, with increased accessibility to MNase digestion and altered plasmid topology. We speculate that if histone abundances were measured in these mutants, they might turn out to have reduced histone content and decreased global nucleosomal occupancy. This might be a fairly common phenotype that was overlooked so far.

The same argument—that decreased histone content must correspond to fewer nucleosomes—also applies to mammalian cells. Genomewide nucleosomal location and occupancy are more difficult to determine, however. The decrease in nucleosome number does not cause an increase in internucleosomal distance (Figures 3B and S1D), but we cannot show rigorously that nucleosome position is conserved, both because of the significant effort and cost of resequencing entire mammalian genomes and because the majority of sequences in mammals are repeated and cannot be assigned to a specific genome position. Thus, even if all mappable nucleosome borders were conserved, non-mappable nucleosome borders (which are the majority in the mammalian genome) might be substantially altered.

### HMG-Box Proteins Are Involved in Genome Chromatinization in Yeast and Mammalian Cells

Despite remaining uncertainties on nucleosomal positions and occupancy in mammalian cells lacking HMGB1, the similarity in phenotype between mammalian and yeast mutants is striking. This suggests that HMG-box proteins might have been involved in determining the number of nucleosomes since the emergence of eukaryotes.

HMGB1 and Nhp6 proteins are functionally similar, and both bend DNA. Since DNA must be bent to wrap around histone octamers and this entails a high energy of activation, DNA-bending proteins might lower the activation energy and provide a chaperone activity on DNA for nucleosome assembly. Expression of a Nhp6 mutant protein unable to bend DNA does not revert the phenotype of Δ*nhp6* cells [32], suggesting that DNA bending is required for the correct chromatinization of the yeast genome. We observed that there is a significant correlation in the yeast genome between the intrinsic propensity to assemble nucleosomes in vitro from high-salt solutions of histones [27] and the nucleosomal occupancy in *nhp6* cells (Figure 8E); this suggests that sites that have an intrinsic difficulty in assembling nucleosomes most need the presence of a DNA-bending protein.

Likewise, we argue that HMGB1 can provide a DNA chaperone activity for nucleosome assembly in mammalian cells: we show that in vitro HMGB1 accelerates nucleosome assembly onto naked DNA.

A chaperone activity only changes the rate of the biological reaction (by lowering the activation energy), and not the equilibrium; thus, we suggest that nucleosome assembly is never at equilibrium in living cells and that the absence of a DNA chaperone will move nucleosome assembly further away from the equilibrium level. This hypothesis has not been formally tested, but we note that the high rate of nucleosome dynamics in living cells makes it very unlikely that equilibrium is ever reached.

We also note that a delay or reduction in nucleosome assembly would lead to a decrease in histone biosynthesis or an increase in histone degradation, or both, via the activity of several feedback control loops [31],[34]. As a consequence, the steady state level of histones in the cell would fall, which is what we observe.

HMG-box proteins can also affect nucleosome dynamics in different ways. For example, we had previously shown that HMGB1 enhances nucleosome remodeling in vitro [10], and the group of Karen Vasquez had shown that histones are not acetylated after DNA damage in the absence of HMGB1 [35]. In yeast, Nhp6 proteins are non-essential components of the FACT complex (in contrast, the absence of the other components Pob3 and Spt16 causes lethality) [7]; the absence of Nhp6 proteins can thus affect nucleosome remodeling associated to transcription. All these activities can affect nucleosomal occupancy directly or indirectly.

### Nucleosome Limitation Correlates with Increased Global Gene Expression and with a Specific Transcriptomic Profile

Nucleosomal occupancy over promoters is a powerful determinant of gene expression. Since we show that genomewide nucleosomal occupancy varies non-uniformly in response to nucleosome depletion, specific transcriptional profiles are expected to ensue. This is exactly what we observe. In both mammalian *Hmgb1*−/− cells and yeast *nhp6* cells, specific genes have increased or decreased expression relative to the rest. These gene-specific effects are distinct from the overall increase in transcription that also takes place and might depend in part on the specific interactions of HMGB1 and Nhp6 proteins with nucleosomes and transcription factors [32]. In turn, changes in gene expression can determine changes in the cell cycle and in cell metabolism, which can lead to further changes in nucleosomal occupancy and gene expression; this makes it difficult to disentangle cause from effect in the phenotype of *nhp6* yeast cells and *Hmgb1*−/− mammalian cells. However, the correlation of transcriptomic profiles in *nhp6* cells (Figure 5E) and in cells where transcription of histone H4 has been shut off [22] suggests that histone depletion is by itself partially responsible for the altered expression of a subset of genes. We therefore propose that overall histone content and the associated modulation of the genomewide and gene-specific nucleosomal landscapes represents a novel layer of epigenetic control of transcription.

## Materials and Methods

### Mouse Embryo Fibroblasts (MEFs)

*Hmgb1*−/− MEFs and their control wild type MEFs were isolated from same-mother embryos deriving from *Hmgb1*+/− crosses [12]. Since *Hmgb1*−/− MEFs accumulate chromosome rearrangements with continuous culturing [14], only cells up to 8 population doublings from embryo isolation were used. MEFs from different mothers gave consistent results in our experiments, and batch-to-batch variation was minimal.

### Quantification of DNA Damage ad DNA Repair

Wild type and *Hmgb1*−/− MEFs were synchronized in G0–G1 by serum starvation for 36 h and gamma-irradiated with 10 Gy by using a ^137^Cs source (Biobeam 2000). The Alkaline Comet Assay was performed as described [15] immediately after irradiation. The extent of DNA damage was measured by calculating the tail moment (Comet Assay II software, Perceptive Instruments).

### Yeast Culture

Wild type and *nhp6* cells [14] were grown in YPD medium at 30°C. For protein quantification cells were arrested in G1 by synthetic alpha-factor pheromone (5 µg/ml) and checked for synchronization by FACS analysis after Sytox staining [36].

### Knockdown in HeLa Cells

Stable HMGB1 knockdown HeLa cells were prepared by transfection with plasmid HMGB1shRNA-pSuperior.puro or, as a mock control, with the empty vector pSuperior.puro (Invitrogen) [37]. Transfected cells were selected with puromycin and single resistant clones were picked, amplified, and analyzed for HMGB1 expression by western blot. Only clones with <10% HMGB1 were used. Transient gene silencing was carried out by performing at 1-d intervals four consecutive transfections of small interfering RNA duplexes (siRNA) against HMGB1 transcript using Lipofectamine 2000 (Invitrogen). siRNA for HMGB1 and for firefly luciferase, as a negative control, were purchased from Dharmacon.

### DNA Quantification by PicoGreen

The Quant-iT PicoGreen dsDNA Kit was used as described by the manufacturer (Invitrogen). Seventy-five µl of diluted PicoGreen reagent were added to each well of a 96-well plate containing either phage λ DNA at known concentrations or the sample (100 µl final volume). After incubation for 5 min at room temperature, fluorescence was measured using a Victor3 plate reader (Ex/Em filters: 485 nm/535 nm, exposure 1.0 s). DNA concentration of the samples was determined by interpolation of the fluorescence intensity values against the standard curve.

### Quantification of Histones by Immunoblot

For western blot analysis, whole-cell extracts were prepared by direct lysis of a defined number of cells in SDS-PAGE sample buffer. DNA from 10^6^ cells was extracted with the DNeasy tissue Kit (Qiagen) and quantified by PicoGreen to confirm cell count. Following electrophoresis, blots were probed with primary antibodies mouse anti-γH2AX (Upstate), rabbit anti-total H2AX (Upstate), rabbit anti-H3 (Abcam), rabbit anti-H2A (Abcam), rabbit anti-H2B (Abcam), rabbit anti-H4 (Abcam), sheep anti-H1 (Abcam), mouse anti-peroxiredoxin-2 (AbFrontier), and mouse or rabbit anti-β-actin (Sigma) for mammalian and yeast, respectively, and visualised using the ECL detection kit (GE Healthcare) or the ECL Plex fluorescent western blotting system (GE Healthcare). Sixteen-bit images were acquired with FLA-9000 (Fuji Film); signals were within the linear part of the dynamic range. Quantification of western blot signals was performed with ImageQuant software (GE Healthcare).

### SILAC-Based MS

SILAC-labelled cells were harvested and mixed 1∶1. Proteins were extracted in SDS-PAGE sample buffer and separated by one-dimensional electrophoresis. Protocols for protein processing and peptide desalting and concentration are described [38],[39]. Peptides were analyzed by nanoflow liquid chromatography on an Agilent 1100 LC system (Agilent Technologies Inc.) coupled to LTQ-FT ultra (Thermo Fisher Scientific). Mass spectrometric data were analyzed for protein identification and peptide quantification with MaxQuant algorithm.

### FACS Analysis of Acridine Orange Stained Cells

2×10^5^ cells resuspended in DMEM with 10% FCS were permeabilized by gently adding 0.4 ml ice-cold permeabilizing solution (0.1% Triton X-100, 80 mM HCl, 150 mM NaCl) and stained with 1.2 ml ice-cold acridine orange staining solution (37 mM citric acid, 126 mM Na_2_HPO_4_, 150 mM NaCl, 1 mM EDTA, 6 µg/ml Acridine Orange). As control, cells were treated with 100 µg/ml RNase A for 10 min after permeabilization. Using the 488 nm laser for excitation, DNA and RNA fluorescence at 530/30 and 610/20 nm, respectively, was recorded with a LSRII flow cytometer (BD Biosciences).

### Slot-Blot Hybridization

Total RNA from 5×10^6^ control and KD HeLa cells was extracted using RNeasy tissue kit (Qiagen). DNA from 10^6^ cells was extracted and quantified by PicoGreen to confirm cell count. Slot blots of total RNA were prepared by denaturing total RNA at 68°C in 1× SSC, 50% formamide, and 5% formaldehyde for 15 min and cooled on ice. Serial 1∶2 dilutions of RNA starting from 400,000 cells were applied to a Nitrocellulose membrane (Protran, Schleicher & Schuell) using a minifold apparatus (Schleicher & Schuell). Total RNA was hybridized to ^32^P end-labeled oligo-dT (25 mer) and to 47S leader sequence oligonucleotide (GGAGACGAGAACGCCTGACACGCACGGCACGGAGCCAGC), to detect polyA^+^ mRNA and 47S rRNA precursor, respectively. The membranes were exposed to an imaging plate (BAS-IP SR 2025, Fuji) and the radiograms were analyzed by BAS-5000 imager (Fuji). Different exposure times were used to obtain densitometric scans (16-bit) in the linear response range. Quantification of RNA slot blot signals was performed with ImageQuant software (GE Healthcare).

### Digestion of Mammalian Chromatin with MNase

Nuclei were isolated as described [40]. Briefly, 10^7^ cells were resuspended in 5 ml ice-cold resuspension buffer (10 mM Tris-Cl pH 7.4, 15 mM NaCl, 60 mM KCl, 1 mM EDTA, 0.1 mM EGTA, 0.15 mM spermine, 0.5 mM spermidine, 1 mM DTT, 0.5 mM PMSF) containing 5% sucrose and 0.1% NP-40. Nuclei were pelletted by centrifugation in a swinging bucket rotor at 100–120 g for 20 min at 4°C through a sucrose “pad” solution (resuspension buffer containing 10% sucrose), washed in ice-cold buffer, and digested with micrococcal nuclease (Sigma-Aldrich cat. N5386) for 5 min at 25°C. DNA was purified with the DNeasy Tissue Kit (Qiagen) and electrophoresed on 1.2% agarose gels.

### Chromatin Assembly Assay

Chromatin was assembled on linear DNA with Chromatin Assembly Kit according to the manufacturer's instructions (Active Motif), with two modifications: we used 0.8 µg histones per 1 µg DNA, and we added either BSA or HMGB1 to a total amount of 10 or 1 µg protein/ml in the titration and time-course experiments, respectively. pGEM-T vector DNA (Promega) was digested with *Sac*II, and the linearized plasmid was purified from gel with Gel Extraction kit (Qiagen). Calf thymus HMGB1 was purified as described [41]. Assembled chromatin was digested for 4 min with the Enzymatic Shearing Cocktail provided in the kit. The residual DNA after digestion was quantified by PicoGreen and electrophoresed on a 1.5% agarose gel. The gel was stained with Syber Safe (Molecular Probes) and scanned with FLA-9000 (Fuji Film).

### Digestion of Yeast Chromatin with MNase

Cells were harvested from a 300 ml culture grown to OD = 0.5 and resupended in 10 ml of a buffer containing 1 M sorbitol, 50 mM Tris-HCl pH 7.5, and 10 mM ß-mercaptoethanol. Thirty million cells were incubated for 10 min at 30°C in the presence of 0.05 mg Zymolyase 100T. The spheroplasts were harvested, resuspended in 3.6 ml nystatin buffer (50 mM NaCl, 1.5 mM CaCl_2_, 20 mM Tris-HCl pH 8.0, 1 M sorbitol, 100 µg/ml nystatin), and divided into 0.4 ml aliquots. The samples were incubated with MNase (from 6.4 units/ml in 2× dilutions) at 25°C for 15 min. The reaction was stopped with 1% SDS, 5 mM EDTA (final concentrations). Samples were incubated with Proteinase K (40 µg/sample) at 56°C for 2 h. DNA was then purified by three phenol/chloroform extractions and ethanol precipitation. RNase treatment (35 µg/sample) was also performed. The DNA was then electrophoresed in 1.2% agarose gels at 1.75 V/cm and visualized by EtBr staining [42].

### Topological Analysis

Circular DNA from yeast cells was prepared by alkaline lysis of spheroplasts [43]. Briefly, cells were treated with Zymolyase as reported for chromatin preparation. Spheroplasts were kept on ice for 10 min with two volumes of 0.2 M NaOH, 1% SDS; 1.5 volumes of 3 M potassium acetate (pH 4.8) were then added and the mixture kept on ice for 45 min. After centrifugation, two volumes of ethanol were added to the supernatant in order to precipitate the circular DNA forms.

2D topoisomer analysis was performed essentially as described [44]. Plasmid DNA, prepared as above, was run on 1% agarose gels in the first direction for 21 h at 60 V, in chloroquine buffer (30 mM NaH_2_PO_4_, 36 mM Tris, 0.5 mM Na-EDTA, 10 µg/ml chloroquine). The gel was then equilibrated in the same buffer containing 30 µg/ml chloroquine for 2 h and run in the perpendicular direction at 40 V in the same buffer with recirculation for 16 h at room temperature. Finally, the gel was blotted and hybridized with the specific probe (plasmid yRp17 digested with EcoRI). The signal of each band in the topoisomer arch was measured with a PhosphorImager.

### High Throughput Sequencing of Mononucleosomal DNA

Chromatin from wild type and *nhp6* yeast cells was prepared as described and digested with MNase. The sample containing mostly mononucleosome-sized DNA (12 units MNase/10^8^ cells, 25°C, 15 min) was run on a 1.2% agarose gel. The mononucleosome band was excised from the gel and used to prepare libraries suitable for the Illumina GA IIx sequencer: the samples were end-repaired, A-tailed, and adapter-ligated (following each step the reaction was cleaned up using the Qiaquick PCR purification kit). Samples were subjected to 12 cycles of PCR amplification before selection of the 250–300 bp fraction from a 2% agarose gel. The library was extracted using the Qiaquick gel extraction kit, quantified using the Quant-iT dsDNA HS Assay, and quality checked using the Agilent bioanalyzer 2100 system. Two biological replicates for both wild type and mutant were sequenced in independent experiments producing 9.65–10.08 M reads at 51 bp. The reads were aligned to the yeast genome using Novoalign producing around 76% unique alignments.

### Nucleosome Mapping and Modelling of Occupancy

High throughput sequence reads were mapped to the yeast genome, and nucleosome positions were extracted using “Template Filtering” [24]. A total of 53,643 positioned nucleosomes were found in the wild type versus 45,441 nucleosomes in *nhp6*. To compare and model the occupancy we considered only nucleosomes that were detected both in wild type and *nhp6* cells. To model occupancy, we assumed that the occupancy of each nucleosome is a hyperbolic function of available histones. The occupancy O of nucleosome *i* is defined by O_i_ = *x*/(*x*+k_i_), where *x* is an unknown parameter of the concentration of available histones and k_i_ is the dissociation constant. In a simple interpretation, *x* can be taken as the concentration of free histones before deposition on DNA, for example during S phase. *x* was set to 1 for the wild type sample and k_i_ was extracted using the measured wild type occupancy. Average occupancy in *nhp6* cells is reduced to 70% of the wild type (based on the measured amount of MNase-resistant DNA); using this parameter, the model returns a concentration of available histones of 0.5 and the occupancy of each single nucleosome. We removed the top 1% highly occupied nucleosomes to avoid overdependence of the model on the extreme values and overvaluation of correlations.

### Gene Expression Profiling

Total RNAs from control and KD HeLa cells were extracted using RNeasy tissue kit (Qiagen). Yeast RNA extraction was performed using standard procedures [43]. RIN (RNA Integrity Number) of each sample was determined with the 2100 Bioanalyzer (Agilent) to assess RNA quality; samples with RIN<8 were discarded. We analyzed four technical replicates for HeLa cells and three biological replicates for yeast cells.

For HeLa cells, total RNA was reverse transcribed with the Illumina TotalPrep RNA Amplification kit (Ambion) and cRNA was generated with a 14 h in vitro transcription reaction. cRNA was then eluted and purified. Washing, staining, and hybridization were performed according to the standard Illumina protocol. RNAs were hybridized to Illumina HumanHT-12 V3.0 expression beadchip; datasets were first quantile normalized (without background subtraction) in BeadStudio v.3.0, then expression data were rescaled by mean centering and standardization; the differentially expressed genes were identified by *t* test, *p*<0.01.

For yeast, Affymetrix Yeast 2.0 chips were hybridized and scanned according to the manufacturer's recommendation. 7G scanner data extracted as .cel files were then analyzed in GeneSpring GX 11.0.1. We used quantile normalization using RMA summarization algorithm with baseline transformation to the median of all samples. We then identified genes with transcript levels differing more than 1.5-fold (*p*<0.05 with Benjamini-Hochberg correction for multiple testing).

Data from mammalian and yeast cells were visualized by hierarchical clustering in TmeV (v. 4.5.1), choosing Euclidean metric and average linkage.

### Data Availability

The complete nucleosome map of wild type and *nhp6* cells is available at http://genome.ucsc.edu/cgi-bin/hgTracks?hgS_doOtherUser=submit&hgS_otherUserName=Assafwe&hgS_otherUserSessionName=NH6PA.

HeLa transcriptome is available on the GEO website with the accession number GSE18721.

Yeast transcriptome is available on the GEO website with the accession number GSE23711.

## Supporting Information

Figure S1 HeLa cells KD for HMGB1 have a reduced amount of histones and altered chromatin compaction. (A) Western blot of HMGB1 and actin in control and KD HeLa cells. (B) Cell cycle distribution of control and KD HeLa cells by propidium iodide staining of DNA. (C) Upper panel: quantification by western blot of histone content from three experiments expressed as KD: control ratio. Error bars, SEM. The reduction of about 20% of both core and linker histones is statistically significant (*p*<0.05, Wilcoxon test). Lower panel: residual (nucleosome-protected) DNA obtained from KD and control HeLa after digestion with increasing MNase concentrations. Error bars, SEM from three biological replicates. (D) Electrophoretic separation and densitometric analysis of DNA samples from 250,000 control and KD HeLa cells after digestion with 0, 0.5, and 2 U/ml of MNase. MW: 100 bp ladder.
(TIF)

Figure S2 SILAC-based quantitative proteomic analysis of control and KD HeLa cells. (A) Scheme of the experimental setup for SILAC, with examples of the MS readout: proteins not responding to HMGB1 depletion show a peptide ratio equal to 1 (orange pairs), whereas peak ratios of protein up- or down-regulated differ from 1 (green and blue pairs). (B) Representative mass spectra for SILAC pairs from various proteins: left column, peptide pairs from reference experiment (control (light) : control (heavy)); right column, peptide pairs from the KD (light) : control (heavy) experiment. Peptide KHPDASVNFSEFSK of HMGB1 has ratio L/H (light/heavy) = 0.1, indicative of 90% depletion efficiency; peptide DAVTYTEHAK from H4 exemplifies histone down-regulation in the light KD sample (L/H = 0.64); peptide LLLEYTDSSYEEK from Glutathione S-transferase Mu 1 exemplifies an up-regulated protein (L/H = 1.87); peptide GLFIIDGK from peroxiredoxin-2 represents proteins with ratio ∼1. (C) Left panel: histogram of log_2_ normalized protein L/H ratios (*n* = 1,818) of the reference proteomes, fitting a normal distribution with a standard deviation of 0.13. Right panel: the wider distribution (SD = 0.37, *n* = 1,813) indicates that a large number of proteins changed their expression level after HMGB1 knockdown. (D) Overlay of the log_2_ protein L/H ratio distributions from the KD : control and control : control experiments.
(TIF)

Figure S3 SILAC analysis of histone variants in control and KD HeLa cells. The box plots represent KD-Light/control-Heavy ratios for the whole proteome (“all,” all peptides) and non-modifiable peptides from histone variants and H4 for comparison (from Figure 2C). Number of peptides: all peptides = 26,823, H2AX = 10, H2AZ = 18, H4 = 81; mean values ± SD: H2AX = 0.718±0.114, H2AZ = 0.704±0.168, H4 = 0.781±0.133). Probabilities are calculated using Wilcoxon test.
(TIF)

Figure S4 *Hmgb1*−/− embryo livers contain a reduced amount of histones. (A) Western blot of serial 1∶2 dilutions starting from 25,000 cells. (B) Ratios of band intensities from the blots in (A) and two other similar experiments. Histone ratios are significantly different from 1 (*p*<0.05, Wilcoxon test), while DNA and actin ratios are not. Error bars represent SEM.
(TIF)

Figure S5 Gene expression analyses in HeLa and yeast cells. Cluster representation of the differentially expressed genes using TmeV software. (A) Four technical replicates of control and KD HeLa cells. (B) Three biological replicates of wild type and *nhp6* cells.
(TIF)

Figure S6 Functional analysis of HeLa KD transcriptome and proteome. Comprehensive Gene ontology (GO) analysis. (A) Transcriptome of HeLa KD versus control. (B) Proteome analysis: (upper panel) light-KD : heavy-control; (lower panel) reference experiment with light-control : heavy-control. GO categories were selected from Cellular Component, Biological Process and Molecular Function GO domains. Significant-responder GO categories, highlighted by an asterisk (*), were selected based on ratio significance B (*p*<0.05). No significant-responder categories were detected in the reference experiment (control versus control).
(TIF)

Figure S7 Functional analysis of transcriptome of *nhp6* cells. Comprehensive Gene ontology (GO) analysis of the transcriptome. GO categories were selected from Cellular Component, Biological Process, and Molecular Function GO domains. Significant-responder GO categories, highlighted by an asterisk (*), were selected based on ratio significance B (*p*<0.05).
(TIF)

Figure S8 Additional information on nucleosome position and modelling of results. (A) Density dot plot showing the simulated relative occupancy per bp of wild type (*x*-axis) versus *nhp6* cells (*y*-axis), assuming that 75% of nucleosomes have the same occupancy, and 25% have very reduced occupancy in *nhp6* cells (not zero, otherwise they would all fall on the *x*-axis). Two subpopulations are immediately apparent. (B) Left: histogram showing the frequency distribution of variation in center positions between nucleosomes from wild type and *nhp6* cells. Right: cumulative frequency distribution of variation in center positions. (C) Distribution of the length of DNA covered by nucleosomes, as identified by template filtering. (D) log_2_ ratios (*nhp6*/wt) of nucleosome occupancy over the entire coding regions of genes grouped in 5 classes (down-regulated to unchanged and up-regulated in *nhp6* cells). Blue dots represent the median log_2_ ratio in the group and the black line shows the 0.25 and 0.75 quartiles. The correlation of −0.07 is statistically significant (*p*<10^−6^). (E) In our model, the occupancy O of nucleosome i is defined by O_i_ = *x*/(*x*+k_i_), where *x* is an unknown parameter of the concentration of available histones and k_i_ is the dissociation constant. *x* was set to 1 for the wild type sample and k_i_ was extracted using the measured wild type occupancies. Average occupancy in *nhp6* cells is reduced to 70% of the wild type (based on the measured amount of MNase-resistant DNA); using this parameter, the model returns a concentration of available histones of 0.5 and the occupancy of each single nucleosome. (F) Representative results obtained from a simple model of statistical positioning. We assume that one of three events can occur at any given time: nucleosome loading (K_on_), nucleosome unloading (K_off_), and nucleosome sliding (K_slide_). Reducing occupancy (lower K_on_ and/or increased K_off_) changes the spacing of nucleosomes (red line) as compared to a wild type fitting (blue line).
(TIF)

Figure S9 The yeast ARS1 locus is more accessible to MNase in *nhp6* cells. (A) High resolution analysis of MNase accessibility of the nucleosome in the ARS1 region (C-domain) in wild type and *nhp6* cells. Ellipses indicate nucleosomes, the filled box the ABF1 binding site. DNA was digested with 1.6 and 3.2 U of MNase when packaged in chromatin, or after deproteinization (lane N), and primer-extended [45] from the labelled oligo ARS1r (position 1024 to 1001, numbering as in [46]). M, molecular weight marker (pBR322 cut with MspI); G, sequencing lane; S, vertical thick lines represent protection from MNase digestion compared to naked DNA; horizontal thin lines hypersensitivity. (B) ChIP of histone H3 in A, B, and C regions of ARS1, shown as *nhp6*/wt enrichment.
(TIF)

## Abbreviations

HMGB1: High Mobility Group Box 1
MEFs: mouse embryo fibroblasts
MS: mass spectrometry
NDR: nucleosome-depleted region
NFR: nucleosome-free region
SILAC: stable isotope labeling with amino acids in cell culture
TSS: transcriptional start site

## References

[1] Kornberg R. D, Thomas J. O (1974). Chromatin structure; oligomers of the histones.. Science.

[2] Li B, Carey M, Workman J. L (2007). The role of chromatin during transcription.. Cell.

[3] Groth A, Rocha W, Verreault A, Almouzni G (2007). Chromatin challenges during DNA replication and repair.. Cell.

[4] van Holde K. E, Rich A (1988). Chromatin;.

[5] Feser J, Truong D, Das C, Carson J. J, Kieft J (2010). Elevated histone expression promotes life span extension.. Mol Cell.

[6] O'Sullivan R. J, Kubicek S, Schreiber S. L, Karlseder J (2010). Reduced histone biosynthesis and chromatin changes arising from a damage signal at telomeres.. Nat Struct Mol Biol.

[7] Stillman D. J (2010). Nhp6: a small but powerful effector of chromatin structure in Saccharomyces cerevisiae.. Biochim Biophys Acta.

[8] Bianchi M. E, Agresti A (2005). HMG proteins: dynamic players in gene regulation and differentiation.. Curr Opin Genet Dev.

[9] Thomas J. O, Travers A. A (2001). HMG1 and 2, and related ‘architectural’ DNA-binding proteins.. Trends Biochem Sci.

[10] Bonaldi T, Längst G, Strohner R, Becker P. B, Bianchi M. E (2002). The DNA chaperone HMGB1 facilitates ACF/CHRAC-dependent nucleosome sliding.. EMBO J.

[11] Gerlitz G, Hock R, Ueda T, Bustin M (2009). The dynamics of HMG protein-chromatin interactions in living cells.. Biochem Cell Biol.

[12] Calogero S, Grassi F, Aguzzi A, Voigtländer T, Ferrier P (1999). The lack of chromosomal protein Hmg1 does not disrupt cell growth, but causes lethal hypoglycaemia in newborn mice.. Nature Genet.

[13] Paull T. T, Carey M, Johnson R. C (1996). Yeast HMG proteins NHP6A/B potentiate promoter-specific transcriptional activation in vivo and assembly of preinitiation complexes in vitro.. Genes Dev.

[14] Giavara S, Kosmidou E, Hande M. P, Bianchi M. E, Morgan A (2005). Yeast Nhp6A/B and mammalian Hmgb1 facilitate the maintenance of genome stability.. Curr Biol.

[15] Hartmann A, Agurell E, Beevers C, Brendler-Schwaab S, Burlinson B (2003). Recommendations for conducting the in vivo alkaline Comet assay. 4th International Comet Assay Workshop.. Mutagenesis.

[16] Elia M. C, Bradley M. O (1992). Influence of chromatin structure on the induction of DNA double strand breaks by ionizing radiation.. Cancer Res.

[17] Ong S. E, Mann M (2006). A practical recipe for stable isotope labeling by amino acids in cell culture (SILAC).. Nat Protoc.

[18] Hodges C, Bintu L, Lubkowska L, Kashlev M, Bustamante C (2009). Nucleosomal fluctuations govern the transcription dynamics of RNA polymerase II.. Science.

[19] Kulaeva O. I, Gaykalova D. A, Pestov N. A, Golovastov V. V, Vassylyev D. G (2009). Mechanism of chromatin remodeling and recovery during passage of RNA polymerase II.. Nat Struct Mol Biol.

[20] Darzynkiewicz Z, Juan G, Srour E. F (2004). Differential staining of DNA and RNA.. Curr Protoc Cytom Chapter.

[21] Bonne-Andrea C, Harper F, Sobczak J, De Recondo A-M (1984). Rat liver HMG1: a physiological nucleosome assembly factor.. EMBO J.

[22] Wyrick J. J, Holstege F. C, Jennings E. G, Causton H. C, Shore D (1999). Chromosomal landscape of nucleosome-dependent gene expression and silencing in yeast.. Nature.

[23] Mavrich T. N, Ioshikhes I. P, Venters B. J, Jiang C, Tomsho L. P (2008). A barrier nucleosome model for statistical positioning of nucleosomes throughout the yeast genome.. Genome Res.

[24] Weiner A, Hughes A, Yassour M, Rando O. J, Friedman N (2010). High-resolution nucleosome mapping reveals transcription-dependent promoter packaging.. Genome Res.

[25] Lowary P. T, Widom J (1998). New DNA sequence rules for high affinity binding to histone octamer and sequence-directed nucleosome positioning.. J Mol Biol.

[26] Segal E, Widom J (2009). What controls nucleosome positions?. Trends Genet.

[27] Kaplan N, Moore I. K, Fondufe-Mittendorf Y, Gossett A. J, Tillo D (2009). The DNA-encoded nucleosome organization of a eukaryotic genome.. Nature.

[28] Vilenchik M. M, Knudson A. G (2003). Endogenous DNA double-strand breaks: production, fidelity of repair, and induction of cancer.. Proc Natl Acad Sci U S A.

[29] Parrinello S, Samper E, Krtolica A, Goldstein J, Melov S (2003). Oxygen sensitivity severely limits the replicative lifespan of murine fibroblasts.. Nat Cell Biol.

[30] Kornberg R. D, Stryer L (1988). Statistical distributions of nucleosomes: nonrandom locations by a stochastic mechanism.. Nucleic Acids Res.

[31] Gunjan A, Paik J, Verreault A (2006). The emergence of regulated histone proteolysis.. Curr Opin Genet Dev.

[32] Dowell N. L, Sperling A. S, Mason M. J, Johnson R. C (2010). Chromatin-dependent binding of the S. cerevisiae HMGB protein Nhp6A affects nucleosome dynamics and transcription.. Genes Dev.

[33] Eriksson P. R, Mendiratta G, McLaughlin N. B, Wolfsberg T. G, Marino-Ramirez L (2005). Global regulation by the yeast Spt10 protein is mediated through chromatin structure and the histone upstream activating sequence elements.. Mol Cell Biol.

[34] Marzluff W. F, Wagner E. J, Duronio R. J (2008). Metabolism and regulation of canonical histone mRNAs: life without a poly(A) tail.. Nat Rev Genet.

[35] Lange S. S, Mitchell D. L, Vasquez K. M (2008). High mobility group protein B1 enhances DNA repair and chromatin modification after DNA damage.. Proc Natl Acad Sci U S A.

[36] Haase S. B (2004). Cell cycle analysis of budding yeast using SYTOX Green.. Curr Protoc Cytom Chapter.

[37] Trisciuoglio L, Bianchi M. E (2009). Several nuclear events during apoptosis depend on caspase-3 activation but do not constitute a common pathway.. PLoS One.

[38] Shevchenko A, Tomas H, Havlis J, Olsen J. V, Mann M (2006). In-gel digestion for mass spectrometric characterization of proteins and proteomes.. Nat Protoc.

[39] Rappsilber J, Mann M, Ishihama Y (2007). Protocol for micro-purification, enrichment, pre-fractionation and storage of peptides for proteomics using StageTips.. Nat Protoc.

[40] Archer T. K, Ricci A. R (1999). Exonuclease III as a probe of chromatin structure in vivo.. Methods Enzymol.

[41] Bonaldi T, Talamo F, Scaffidi P, Ferrera D, Porto A (2003). Monocytic cells hyperacetylate chromatin protein HMGB1 to redirect it towards secretion.. EMBO J.

[42] Venditti S, Camilloni G (1994). In vivo analysis of chromatin following nystatin-mediated import of active enzymes into Saccharomyces cerevisiae.. Mol Gen Genet.

[43] Di Mauro E, Camilloni G, Verdone L, Caserta M (1993). DNA topoisomerase I controls the kinetics of promoter activation and DNA topology in Saccharomyces cerevisiae.. Mol Cell Biol.

[44] Peck L. J, Wang J. C (1983). Energetics of B-to-Z transition in DNA.. Proc Natl Acad Sci U S A.

[45] Di Felice F, Chiani F, Camilloni G (2008). Nucleosomes represent a physical barrier for cleavage activity of DNA topoisomerase I in vivo.. Biochem J.

[46] Venditti P, Costanzo G, Negri R, Camilloni G (1994). ABFI contributes to the chromatin organization of Saccharomyces cerevisiae ARS1 B-domain.. Biochim Biophys Acta.

