# Supplementary figures and images for: Substantial Histone Reduction Modulates Genomewide Nucleosomal Occupancy and Global Transcriptional Output
Source: PLoS Biol. 2011 Jun 28;9(6):e1001086. doi: 10.1371/journal.pbio.1001086 (PMC3125158; doi:10.1371/journal.pbio.1001086)

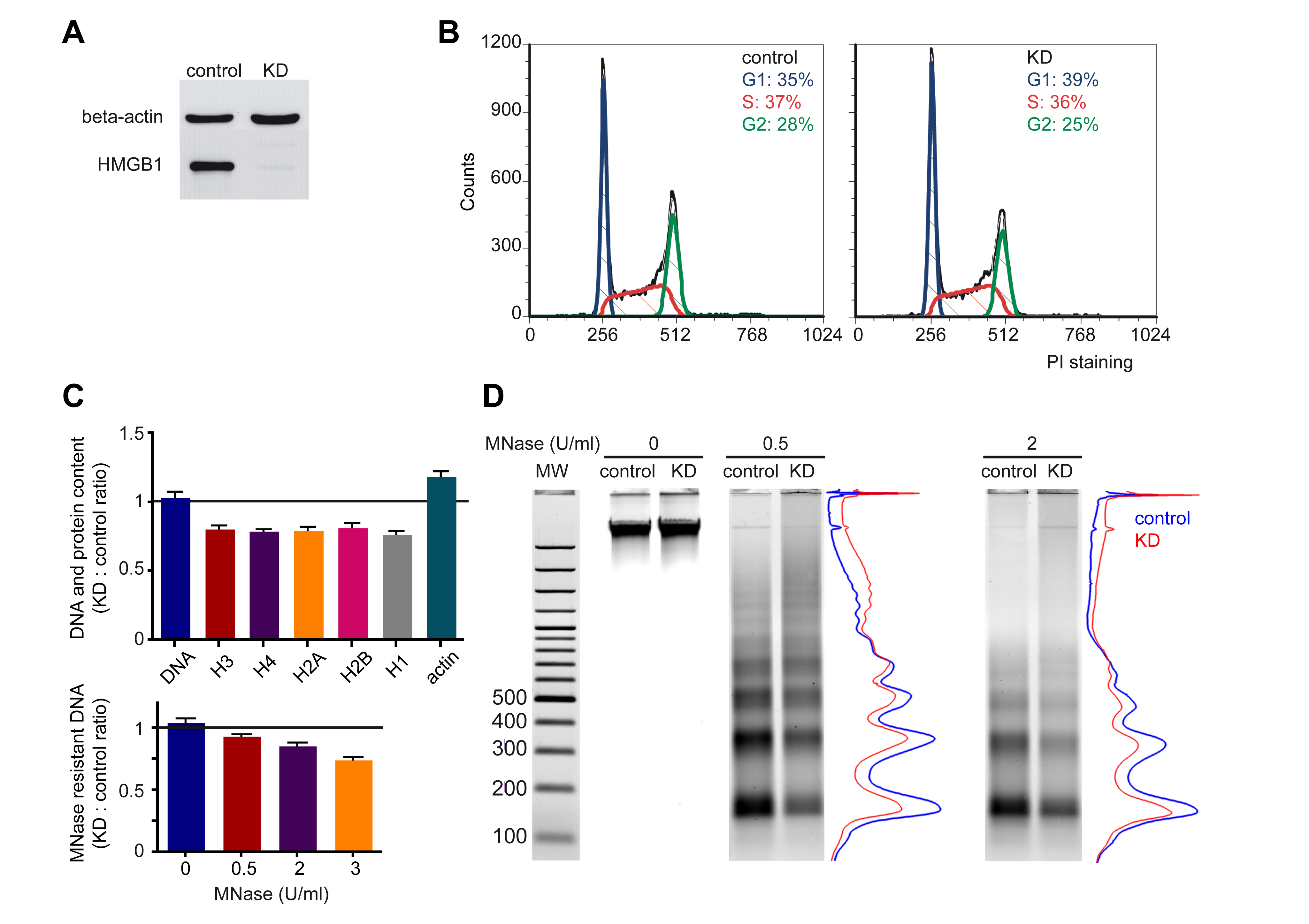

Supplement: Figure S1 — HeLa cells KD for HMGB1 have a reduced amount of histones and altered chromatin compaction. (A) Western blot of HMGB1 and actin in control and KD HeLa cells. (B) Cell cycle distribution of control and KD HeLa cells by propidium iodide staining of DNA. (C) Upper panel: quantification by western blot of histone content from three experiments expressed as KD: control ratio. Error bars, SEM. The reduction of about 20% of both core and linker histones is statistically significant (p<0.05, Wilcoxon test). Lower panel: residual (nucleosome-protected) DNA obtained from KD and control HeLa after digestion with increasing MNase concentrations. Error bars, SEM from three biological replicates. (D) Electrophoretic separation and densitometric analysis of DNA samples from 250,000 control and KD HeLa cells after digestion with 0, 0.5, and 2 U/ml of MNase. MW: 100 bp ladder. (TIF) [file pbio.1001086.s001.tif]

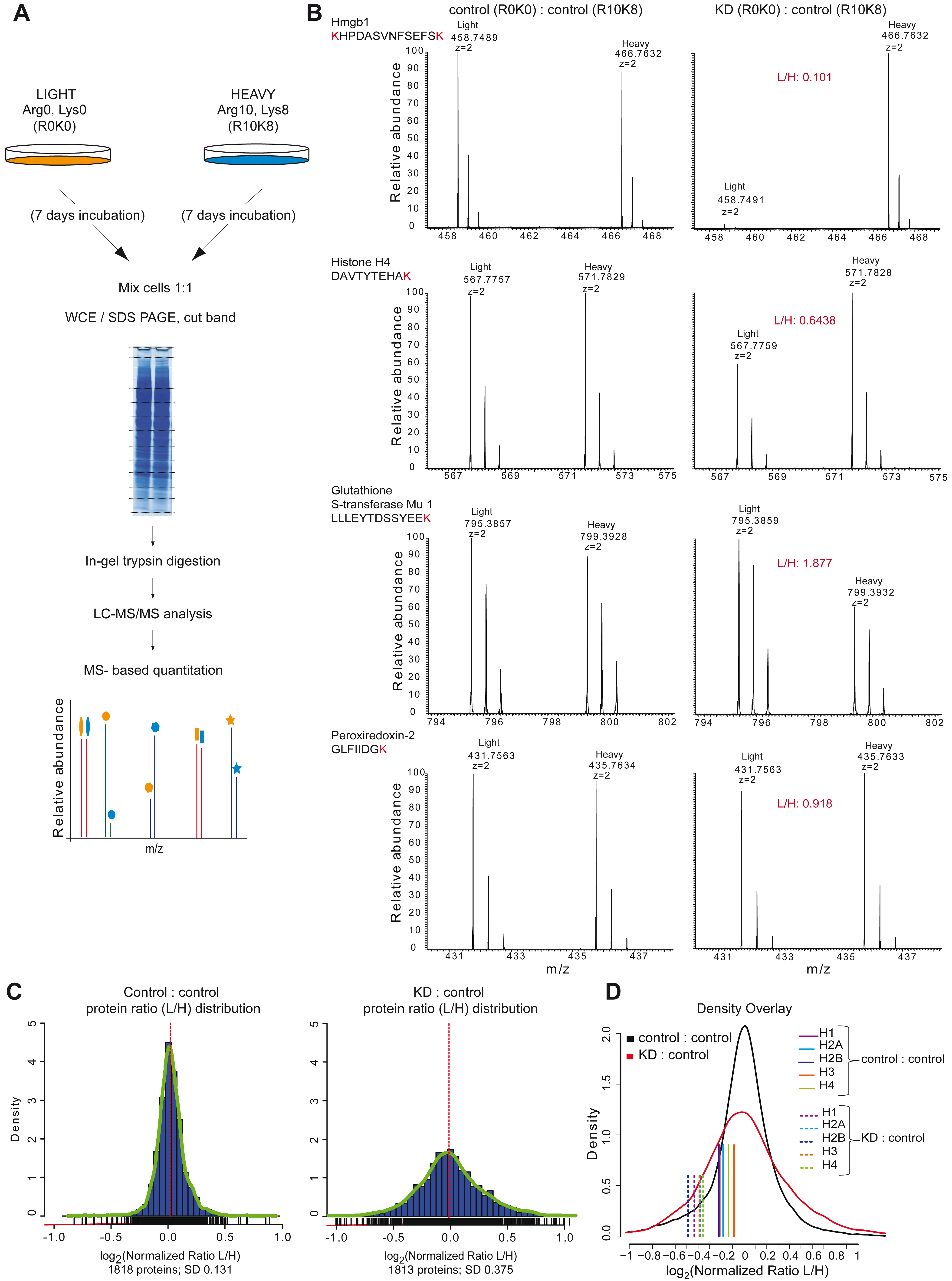

Supplement: Figure S2 — SILAC-based quantitative proteomic analysis of control and KD HeLa cells. (A) Scheme of the experimental setup for SILAC, with examples of the MS readout: proteins not responding to HMGB1 depletion show a peptide ratio equal to 1 (orange pairs), whereas peak ratios of protein up- or down-regulated differ from 1 (green and blue pairs). (B) Representative mass spectra for SILAC pairs from various proteins: left column, peptide pairs from reference experiment (control (light) : control (heavy)); right column, peptide pairs from the KD (light) : control (heavy) experiment. Peptide KHPDASVNFSEFSK of HMGB1 has ratio L/H (light/heavy) = 0.1, indicative of 90% depletion efficiency; peptide DAVTYTEHAK from H4 exemplifies histone down-regulation in the light KD sample (L/H = 0.64); peptide LLLEYTDSSYEEK from Glutathione S-transferase Mu 1 exemplifies an up-regulated protein (L/H = 1.87); peptide GLFIIDGK from peroxiredoxin-2 represents proteins with ratio ∼1. (C) Left panel: histogram of log2 normalized protein L/H ratios (n = 1,818) of the reference proteomes, fitting a normal distribution with a standard deviation of 0.13. Right panel: the wider distribution (SD = 0.37, n = 1,813) indicates that a large number of proteins changed their expression level after HMGB1 knockdown. (D) Overlay of the log2 protein L/H ratio distributions from the KD : control and control : control experiments. (TIF) [file pbio.1001086.s002.tif]

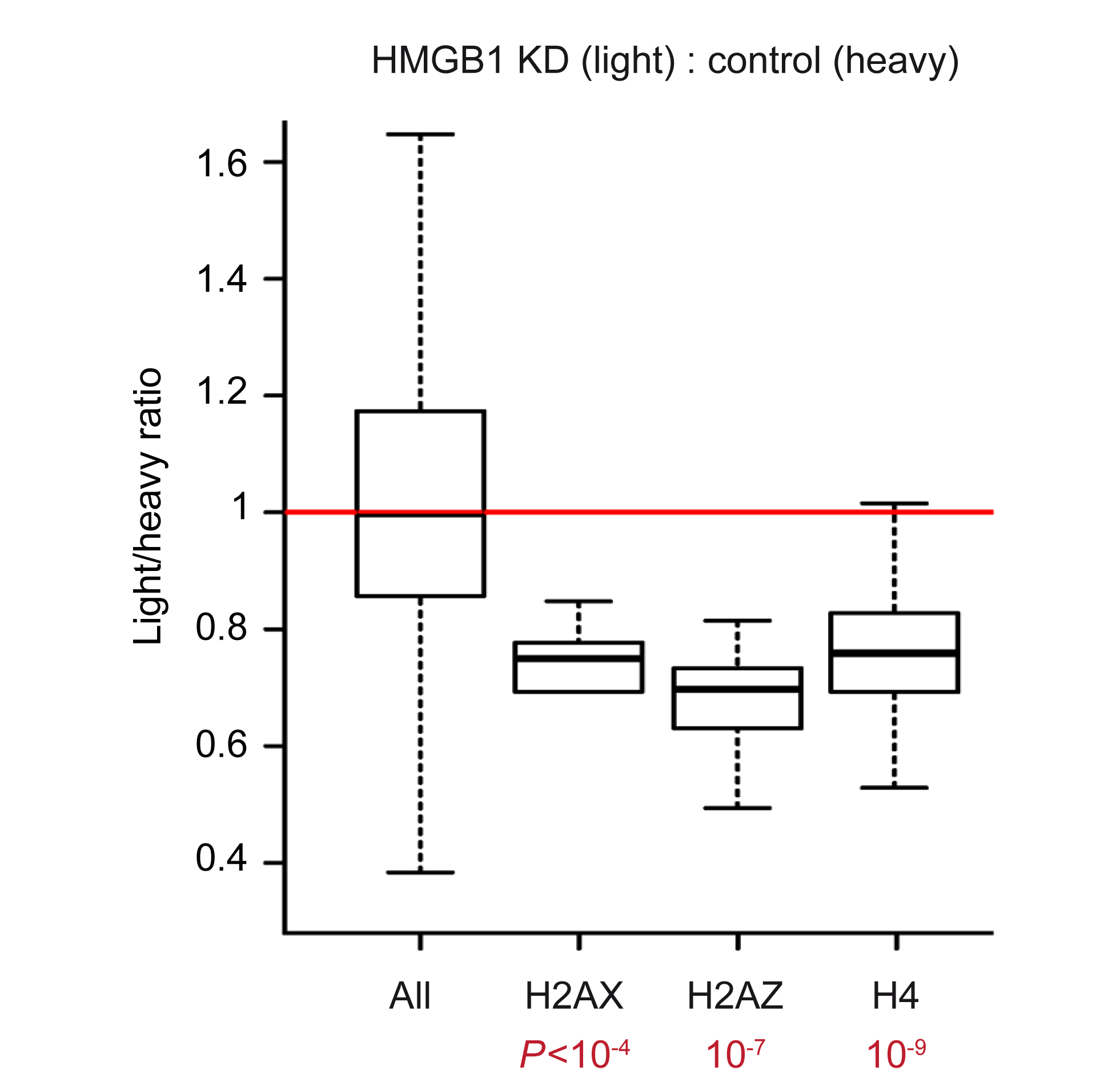

Supplement: Figure S3 — SILAC analysis of histone variants in control and KD HeLa cells. The box plots represent KD-Light/control-Heavy ratios for the whole proteome (“all,” all peptides) and non-modifiable peptides from histone variants and H4 for comparison (from Figure 2C). Number of peptides: all peptides = 26,823, H2AX = 10, H2AZ = 18, H4 = 81; mean values ± SD: H2AX = 0.718±0.114, H2AZ = 0.704±0.168, H4 = 0.781±0.133). Probabilities are calculated using Wilcoxon test. (TIF) [file pbio.1001086.s003.tif]

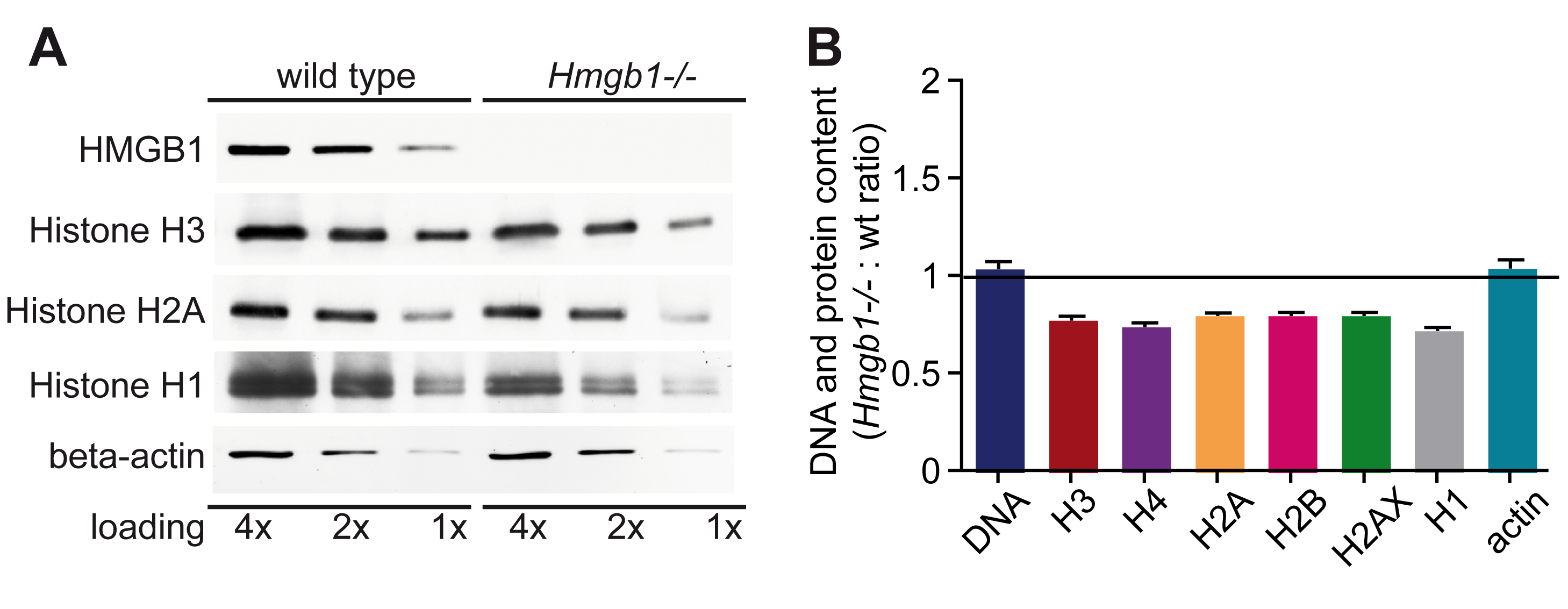

Supplement: Figure S4 — Hmgb1−/− embryo livers contain a reduced amount of histones. (A) Western blot of serial 1∶2 dilutions starting from 25,000 cells. (B) Ratios of band intensities from the blots in (A) and two other similar experiments. Histone ratios are significantly different from 1 (p<0.05, Wilcoxon test), while DNA and actin ratios are not. Error bars represent SEM. (TIF) [file pbio.1001086.s004.tif]

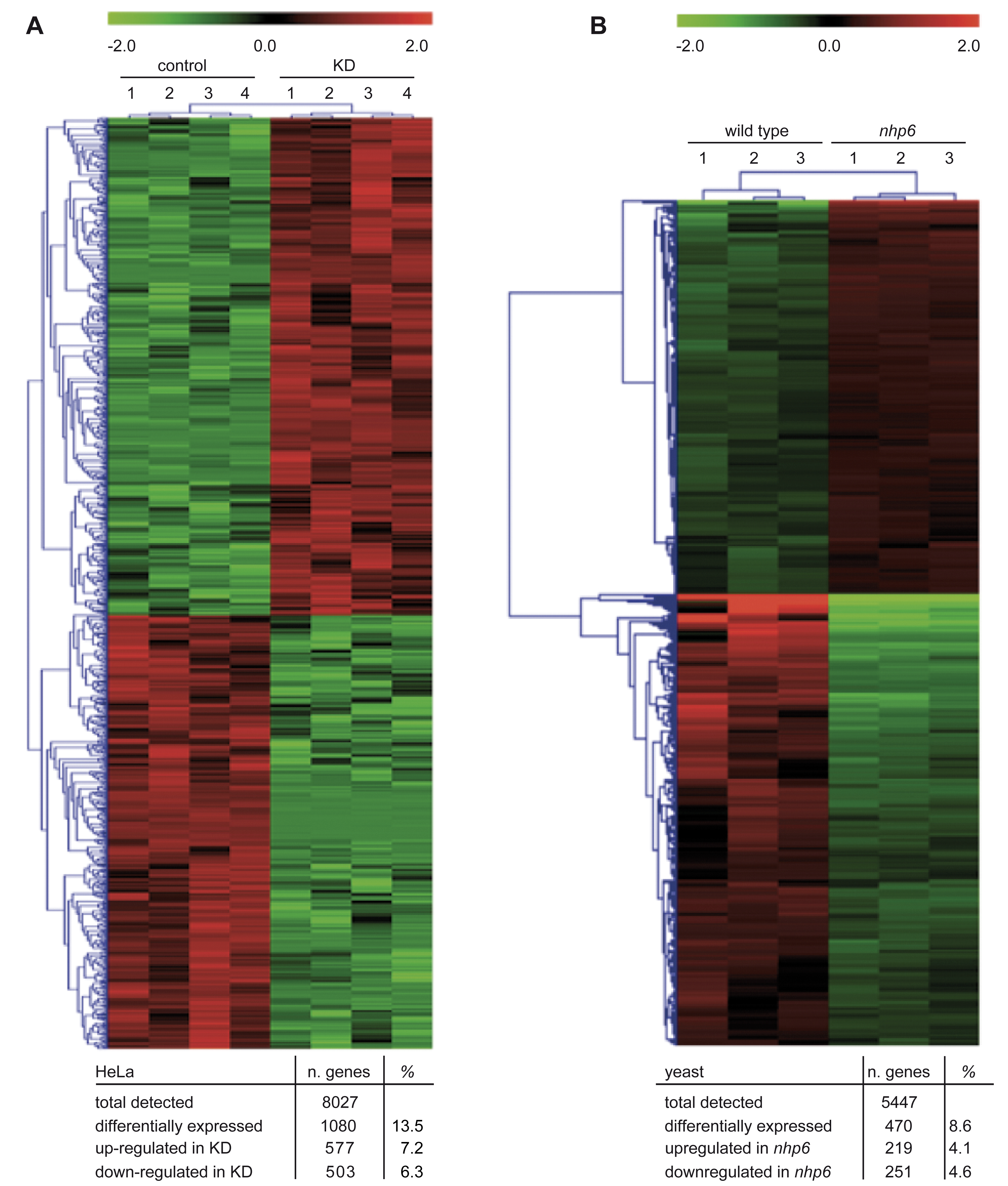

Supplement: Figure S5 — Gene expression analyses in HeLa and yeast cells. Cluster representation of the differentially expressed genes using TmeV software. (A) Four technical replicates of control and KD HeLa cells. (B) Three biological replicates of wild type and nhp6 cells. (TIF) [file pbio.1001086.s005.tif]

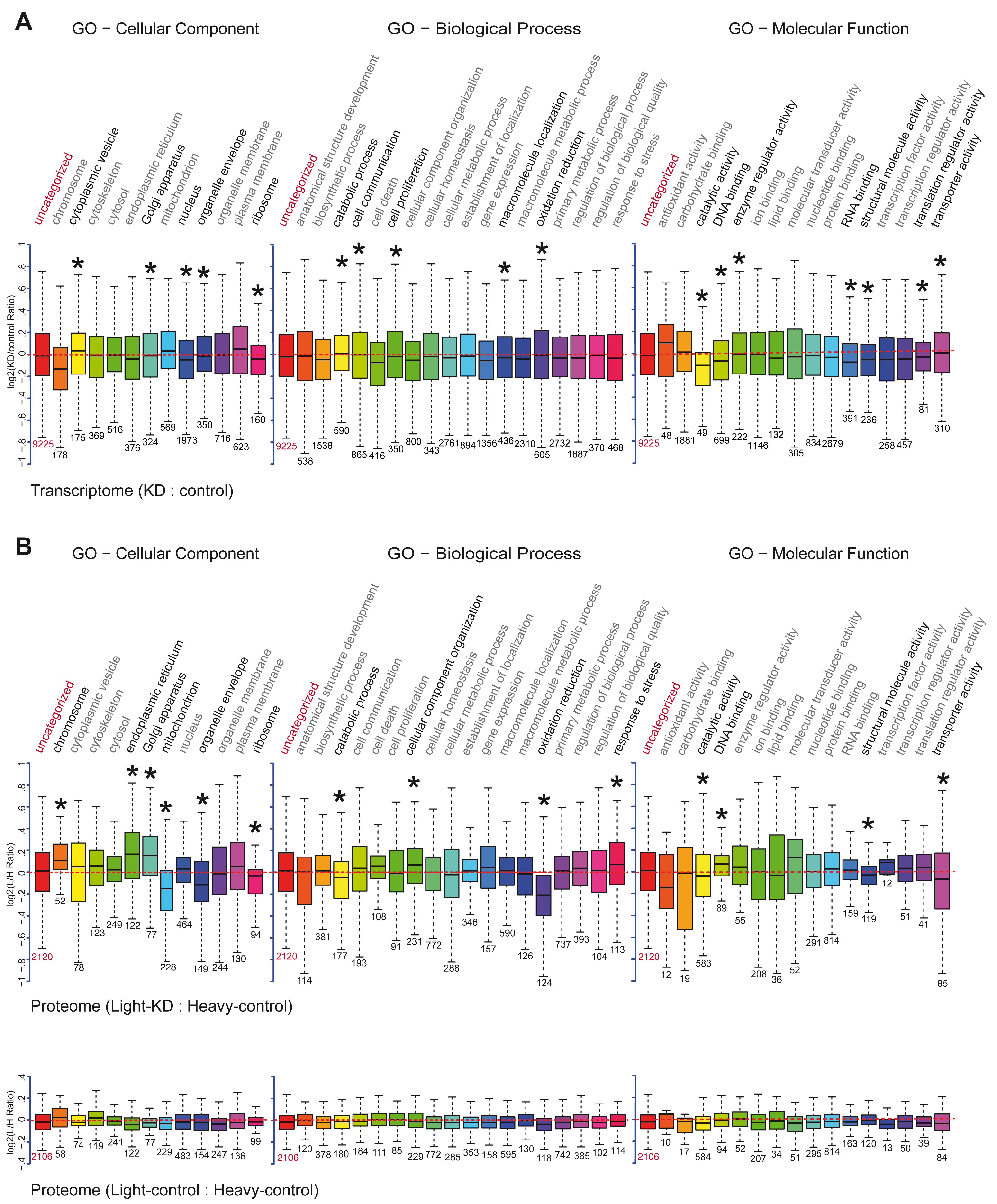

Supplement: Figure S6 — Functional analysis of HeLa KD transcriptome and proteome. Comprehensive Gene ontology (GO) analysis. (A) Transcriptome of HeLa KD versus control. (B) Proteome analysis: (upper panel) light-KD : heavy-control; (lower panel) reference experiment with light-control : heavy-control. GO categories were selected from Cellular Component, Biological Process and Molecular Function GO domains. Significant-responder GO categories, highlighted by an asterisk (*), were selected based on ratio significance B (p<0.05). No significant-responder categories were detected in the reference experiment (control versus control). (TIF) [file pbio.1001086.s006.tif]

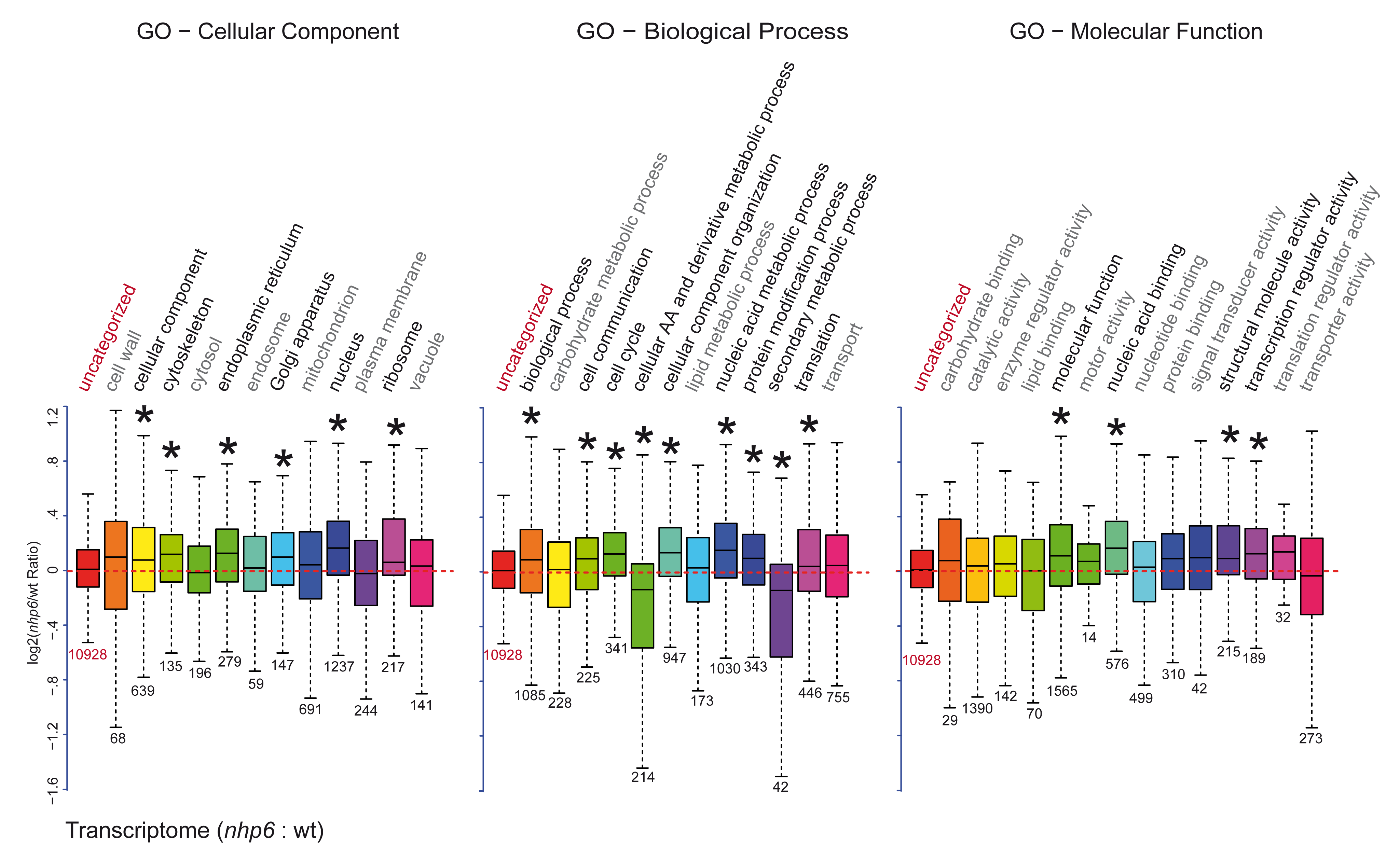

Supplement: Figure S7 — Functional analysis of transcriptome of nhp6 cells. Comprehensive Gene ontology (GO) analysis of the transcriptome. GO categories were selected from Cellular Component, Biological Process, and Molecular Function GO domains. Significant-responder GO categories, highlighted by an asterisk (*), were selected based on ratio significance B (p<0.05). (TIF) [file pbio.1001086.s007.tif]

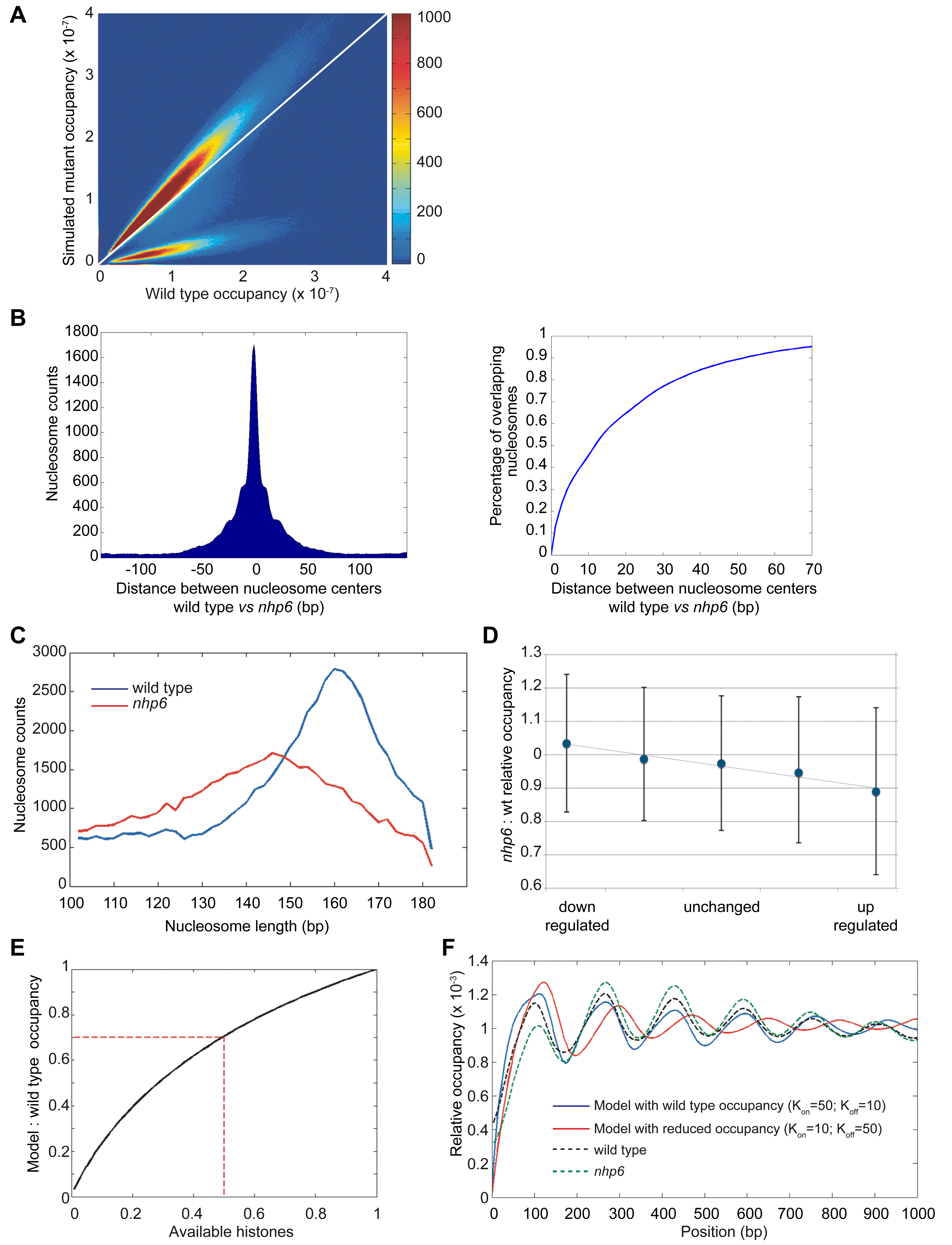

Supplement: Figure S8 — Additional information on nucleosome position and modelling of results. (A) Density dot plot showing the simulated relative occupancy per bp of wild type (x-axis) versus nhp6 cells (y-axis), assuming that 75% of nucleosomes have the same occupancy, and 25% have very reduced occupancy in nhp6 cells (not zero, otherwise they would all fall on the x-axis). Two subpopulations are immediately apparent. (B) Left: histogram showing the frequency distribution of variation in center positions between nucleosomes from wild type and nhp6 cells. Right: cumulative frequency distribution of variation in center positions. (C) Distribution of the length of DNA covered by nucleosomes, as identified by template filtering. (D) log2 ratios (nhp6/wt) of nucleosome occupancy over the entire coding regions of genes grouped in 5 classes (down-regulated to unchanged and up-regulated in nhp6 cells). Blue dots represent the median log2 ratio in the group and the black line shows the 0.25 and 0.75 quartiles. The correlation of −0.07 is statistically significant (p<10−6). (E) In our model, the occupancy O of nucleosome i is defined by Oi = x/(x+ki), where x is an unknown parameter of the concentration of available histones and ki is the dissociation constant. x was set to 1 for the wild type sample and ki was extracted using the measured wild type occupancies. Average occupancy in nhp6 cells is reduced to 70% of the wild type (based on the measured amount of MNase-resistant DNA); using this parameter, the model returns a concentration of available histones of 0.5 and the occupancy of each single nucleosome. (F) Representative results obtained from a simple model of statistical positioning. We assume that one of three events can occur at any given time: nucleosome loading (Kon), nucleosome unloading (Koff), and nucleosome sliding (Kslide). Reducing occupancy (lower Kon and/or increased Koff) changes the spacing of nucleosomes (red line) as compared to a wild type fitting (blue line). (TIF) [file pbio.1001086.s008.tif]

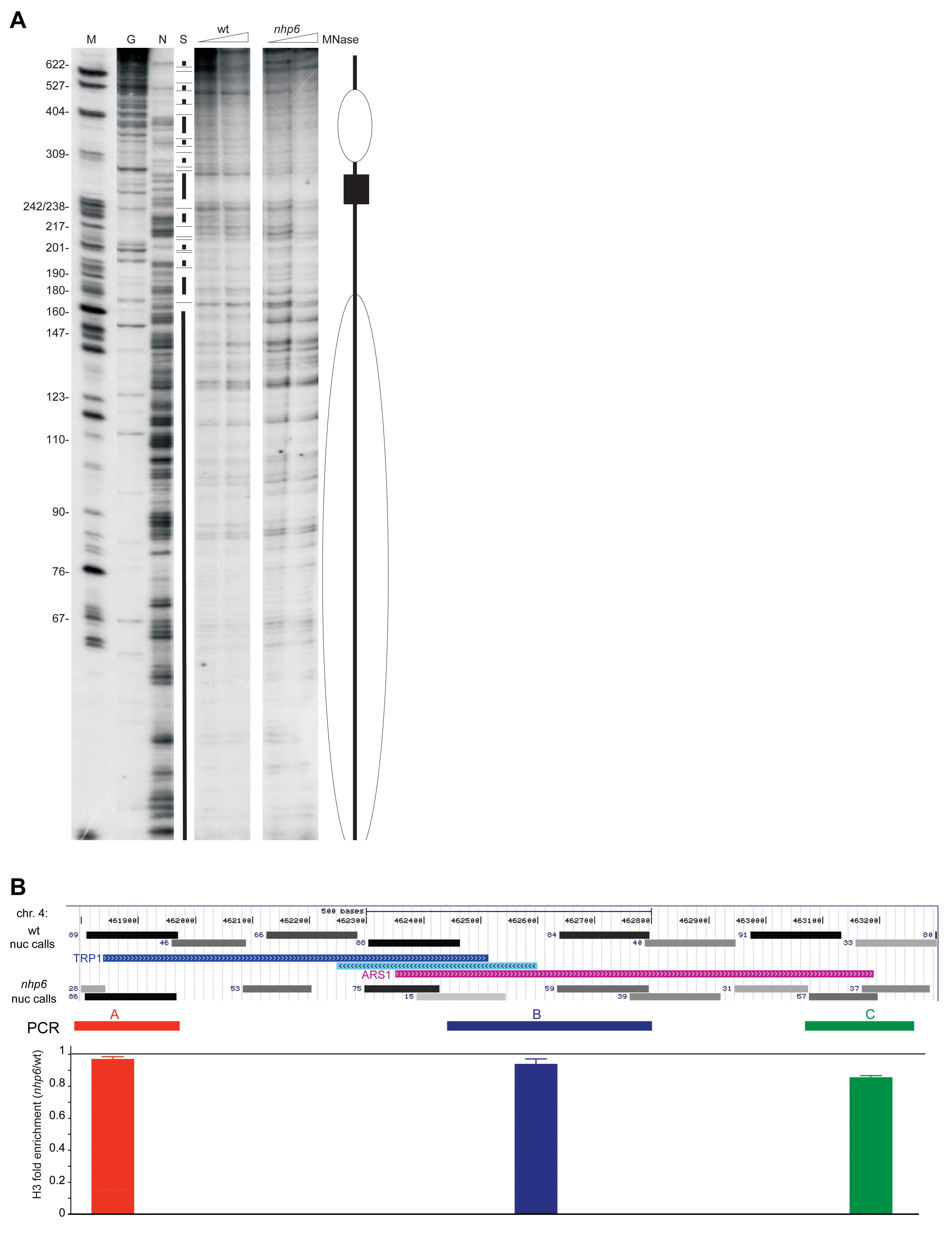

Supplement: Figure S9 — The yeast ARS1 locus is more accessible to MNase in nhp6 cells. (A) High resolution analysis of MNase accessibility of the nucleosome in the ARS1 region (C-domain) in wild type and nhp6 cells. Ellipses indicate nucleosomes, the filled box the ABF1 binding site. DNA was digested with 1.6 and 3.2 U of MNase when packaged in chromatin, or after deproteinization (lane N), and primer-extended [45] from the labelled oligo ARS1r (position 1024 to 1001, numbering as in [46]). M, molecular weight marker (pBR322 cut with MspI); G, sequencing lane; S, vertical thick lines represent protection from MNase digestion compared to naked DNA; horizontal thin lines hypersensitivity. (B) ChIP of histone H3 in A, B, and C regions of ARS1, shown as nhp6/wt enrichment. (TIF) [file pbio.1001086.s009.tif]
